# Quantitative assessment of the impact of cryopreservation on human bone marrow-derived mesenchymal stem cells: up to 24 h post-thaw and beyond

**DOI:** 10.1186/s13287-020-02054-2

**Published:** 2020-12-14

**Authors:** Soukaina Bahsoun, Karen Coopman, Elizabeth C. Akam

**Affiliations:** 1 School of Sport, Exercise and Health Sciences, Loughborough University, Loughborough, Leicestershire LE11 3TU UK; 2 Centre for Biological Engineering, Loughborough University, Loughborough, Leicestershire LE11 3TU UK

**Keywords:** Cell therapy, Cryopreservation, Freezing, Thawing, Quantitative, Human bone marrow-derived mesenchymal stem cells

## Abstract

**Background:**

The effects of cryopreservation on human bone marrow-derived mesenchymal stem cells (hBM-MSCs) are still ill-defined. In this study, a quantitative approach was adopted to measure several post-thaw cell attributes in order to provide an accurate reflection of the freezing and thawing impact.

**Methods:**

Fresh and cryopreserved passage-matched cells from three different donors were discretely analysed and compared for their viability, apoptosis level, phenotypic marker expression, metabolic activity, adhesion potential, proliferation rate, colony-forming unit ability (CFUF) and differentiation potentials.

**Results:**

The results of this study show that cryopreservation reduces cell viability, increases apoptosis level and impairs hBM-MSC metabolic activity and adhesion potential in the first 4 h after thawing. At 24 h post-thaw, cell viability recovered, and apoptosis level dropped but metabolic activity and adhesion potential remained lower than fresh cells. This suggests that a 24-h period is not enough for a full recovery. Beyond 24 h post-thaw, the observed effects are variable for the three cell lines. While no difference is observed in the pre- and post-cryopreservation proliferation rate, cryopreservation reduced the CFUF ability of two of the cell lines and variably affected the adipogenic and osteogenic differentiation potentials of the three cell lines.

**Conclusion:**

The data collected in this study clearly show that fresh and cryopreserved hBM-MSCs are different, and these differences will inevitably introduce variabilities to the product and process development and subsequently imply financial losses. In order to avoid product divergence pre- and post-cryopreservation, effective strategies to mitigate freezing effects must be developed and implemented.

## Introduction

The cell therapy industry is expected to provide life-changing treatments. To become publicly available, these treatments must be cost-effective, delivered to a large number of patients and conveniently stored as on-the-shelf products for when needed. The current best scenario for product distribution is to cryopreserve, deliver and store. Cells are then to be thawed and injected into a patient within hours [1].

Manufacturing autologous or allogeneic therapies requires a significant expansion process in order to produce enough cells to treat one patient multiple times and/or to treat many patients. The main challenge in manufacturing cell therapies is to ensure that cellular characteristics and function are not affected in the end-to-end process. Cell isolation, characterisation, preservation and storage are the integral parts of the upstream as well as downstream processes, whether storage after harvest of starting biopsy material, creation of working/master cell banks, or shipment, delivery and storage as off-the-shelf products. As it currently stands, storage and preservation processes, particularly cryopreservation, are unoptimised and often imperfect. Current cryopreservation protocols still affect bone marrow-derived mesenchymal stem cell (BM-MSC) function in several ways [2], and this fact should be accounted for considering the tight regulations within the pharmaceutical and medicinal product industries. Massie et al. [3] described cryopreservation as a bottleneck in the manufacturing and clinical delivery of regenerative medicine. Yet, for regenerative medicine to bring significant therapeutic benefit to large numbers of people, cryopreservation should deliver functional products at a large scale while complying with Good Manufacturing Processes [3].

Despite presenting a bottleneck within the regenerative medicine pipeline, the benefits of cryopreservation to the cell therapy industry cannot be underestimated. It allows cell transport, the generation of cell banks, the availability of off-the-shelf cell therapy supply and time for quality control testing [4–6]. Moreover, stored therapeutic doses could be potentially used for a scheduled as well for urgent treatments [7]. However, given the unoptimised state of the process, ‘the “cold truth” is that cryopreservation is not a process change that can be taken lightly—the stakes are simply too high’ [8].

Cryopreservation is a common practice in many research institutes owing to its successful application to many cell types and the high levels of cellular recovery that appear to be maintained on thawing. However, any negative impact of the process on the viability and function of cells would be weighed differently in the scenario of a cell therapy development [9]. The state of the final product (fresh or frozen) should be considered very early on in the process development and integrated into the business plan [9]. If a cryopreservation process is implemented, a full and comprehensive understanding of this step must be attained in order to identify the cell quality attributes which may be affected. Moreover, long-term assessment (> 24 h) is necessary to fully evaluate the final product and its critical to quality attributes [9].

Human BM-MSCs are an important tool for the cell therapy industry; however, the various and current cryopreservation protocols affect these cells in several ways such as lower post-thaw viability and metabolic activity [2]. In addition, the effects of cryopreservation on attachment and migration ability, genomic stability and paracrine function are still ill-defined [2]. Several studies have proposed that a 24-h recovery period may take MSCs back to a better functional state [10–13]. However, the detailed behaviour of BM-MSCs in the first 24 h post-thaw and beyond is not fully understood.

The aim of the current study is to quantitatively investigate the impact of a standard cryopreservation procedure on hBM-MSCs in the first 24 h post-thaw and beyond. Studies across the first 24 h post-thaw are vital for cell therapies intended for infusion only hours after retrieval from cryostorage. In fact, more than one third of the current MSC-based clinical trials use cryopreserved cells [11, 14]. It is well known that the freezing-thawing process causes viability loss and induces apoptosis in cells, and the latter takes some time to manifest. Therefore, viability and apoptosis levels were assessed immediately (0 h), 2 h, 4 h and 24 h post-thaw. Moreover, phenotypic surface marker expression, metabolic activity and adhesion potential were assessed concomitantly at the same time points. These chosen time points reflect the time point for intravenous injection upon thawing and up to 4 h post-thaw, a time frame important for assessing delayed onset apoptosis and for reflecting delays in operation theatre (either foreseen or unforeseen procedural timings). In order to assess long-term (beyond 24 h) cellular behaviour in response to the cold journey, hBM-MSC proliferation, colony-forming ability, and osteogenic and adipogenic differentiation potentials were measured before freezing and after thawing. To our knowledge, this is the first study which quantitatively measures all these MSC attributes in three different hBM-MSC cell lines originating from three donors, with each cell line assessed discretely. Comparison between passage-matched fresh and cryopreserved conditions for each cell line was conducted in order to report any changes caused by cryopreservation.

## Materials and methods

### Mesenchymal stem cell (MSC) lines

Three different bone marrow aspirates from healthy donors were purchased from Lonza (USA). hBM-MSCs were isolated, and cells from passage 0 or 1 were cryopreserved in foetal bovine serum (FBS) (RMBIO, US) supplemented with 10% (v/v) dimethysulphoxide (DMSO) (Sigma, UK). The three cell lines were arbitrarily designated as M4, M6 and M7. Please refer to Table 1 for nomenclature and donor information.

**Table 1:** hBM-MSC nomenclature and donor information

Nomenclature	Donor				Supplier
	Age (years)	Gender	Ethnicity	Lot number	
**M4**	20–29	1	Hispanic	0000327825	Lonza (USA)
**M6**	20–29	1	Caucasian	0F3660	Lonza (USA)
**M7**	15–25	1	Caucasian	070834A	Lonza (USA)

### Human BM-MSC culture

Cells were seeded at 5000 cells per square centimetre in Dulbecco’s modified Eagle’s medium (DMEM) (Gibco, UK) supplemented with 10% (v/v) FBS (RMBIO, USA) in Nunc™ cell culture-treated flasks with filter caps (Fisher Scientific). All cultures were maintained at 37 °C and 5% CO_2_ in a humidified incubator. The medium was completely changed at day 3. At day 6 in culture, growth medium was aspirated, and cells were washed with 1× phosphate-buffered saline (PBS), no calcium and no magnesium (Gibco, UK), then detached with 0.25% (w/v) trypsin-EDTA (Gibco, UK) for 3 min, counted (using the Countess Automated Cell Counter (Thermo Fisher Scientific, UK)) and re-seeded at 5000 cells per square centimetre. For all assays, cells at passage 4 (P4) were used. At the end of P4, half of the cells were immediately used for seeding the continuous culture condition assays (fresh) and the other half were frozen for the cryopreserved condition.

### Cryopreservation and thawing of hBM-MSCs

At the end of P4, cells were detached, centrifuged at 200*g* (rpm 1200) and counted. According to the cell number, the cells were re-suspended at 1 × 10^6^ cells per millilitre in FBS supplemented with 10% DMSO (v/v) (Sigma, UK). One millilitre of cell suspension was transferred to each vial (Corning cryogenic vials, internal thread, 2 mL capacity). Vials were kept in Mr Frosty (Nalgene cryogenic freezing container filled with 100% isopropyl alcohol) in a − 80 °C freezer for 24 h to cool at a rate of − 1 °C/min. After 24 h, the vials were transferred to liquid nitrogen (LN_2_) for a minimum of 1 week.

For thawing, vials were removed from LN_2_ and immediately placed through a floating mat in a water bath at 40 °C for exactly 1 min to maintain consistency in the thawing process across all the vials used in all experiments. Next, cells were added to a fresh warm complete medium (9 mL) to dilute DMSO and centrifuged at 200*g* for 5 min at room temperature. The supernatant was discarded, and cells were then re-suspended in a complete fresh medium, counted and used for assays. Thawed cells were immediately used for the 0-h assays or incubated in a fresh complete medium in a humidified incubator for 2, 4 or 24 h and then used for the assays.

### Human BM-MSC immunophenotyping

The expression of the surface markers was assessed by flow cytometry using the MSC phenotyping kit (human) (Miltenyi Biotec, UK) according to the manufacturer’s instructions. The kit confirms compliance with the International Society for Cellular Therapy (ISCT) criteria [15]. Positive markers stained for are CD105 linked to PE, CD90 linked FITC and CD73 linked to APC. Negative markers stained for are CD14, CD20, CD34, CD45 and HLA-DR all linked to PerCP. For fresh cells, the analysis was done at the end of P4 only. Cryopreserved cells were analysed immediately (0 h), 2 h, 4 h and 24 h after thawing. The flow cytometer was compensated for hBM-MSCs according to the manufacturer’s instructions. About 5 × 10^5^ cells were suspended in 100 μL flow cytometry buffer. Then, 10 μL of human MSC phenotyping cocktail and 10 μL of human anti-HLA-DR-PerCP were added and mixed well by pipetting. After a 10-min incubation at 5 °C in the dark, cells were re-suspended in 1 mL buffer then centrifuged, supernatant removed and then finally re-suspended in 500 μL fresh buffer for analysis. For control purposes, unstained samples and the corresponding isotype controls (provided with the kit) were also prepared and analysed. All samples were analysed using the BD Accuri C6 (BD Biosciences, UK). A total of 100,000 events were collected for each analysis. The collected data was analysed using the BD Accuri C6 plus software.

### Viability and apoptosis assay

The viability and percentage of apoptotic cells were assessed using the FITC Annexin V apoptosis detection kit with 7-AAD (BioLegend, UK) according to the manufacturer’s instructions. Fresh cells were only analysed at the end of P4. Cryopreserved cells were analysed immediately (0 h), 2 h, 4 h and 24 h after thawing. For the staining protocol, about 3 × 10^5^ cells were suspended in 100 μL Annexin V binding buffer. Then, 5 μL of FITC annexin V and 5 μL of 7AAD viability staining solution were added. Cells were incubated at room temperature in the dark for 15 min. After that, cells were analysed using the DB Accuri C6. A total of 100,000 events were collected for each analysis. All data was analysed using the BD Accuri C6 plus software.

### WST-1 assay

A total of 30,000 cells per well were seeded in 100 μL of complete growth media in 96-well plates. Ten microlitres of warm WST-1 solution (Sigma, UK) was added to each well at 0 h, 2 h, 4 h or 24 h after seeding. Plates were then incubated for further 2 h at 37 °C in a humidified incubator. After incubation, the absorbance was measured at 450 nm using the Varioskan Flash spectral scanning multimode reader (Thermo Scientific, UK).

### Adhesion assay

An adhesion assay based on a published protocol was developed [16]. A total of 100,000 cells per well were seeded in 100 μL of complete growth media in 96-well plates. Plates were incubated for 1 h, 2 h, 4 h or 24 h then stained with 0.1% (w/v) crystal violet (Sigma, UK) solution in ethanol. The staining process consisted of aspirating the growth media, washing with PBS, fixing with 4% (w/v) paraformaldehyde solution (Fisher Scientific, UK) for 10 min, washing with PBS, incubating with crystal violet solution for 10 min then washing twice with distilled water. The plates were then tapped on towels before reading the absorbance at 550 nm using the Varioskan Flash spectral scanning multimode reader (Thermo Scientific, UK).

### Cell proliferation assay

To determine the growth of fresh and cryopreserved cells of each cell line, six T25 flasks at a density of 5000 cells per square centimetre were seeded. At days 1 to 6 each, one flask was removed from the incubator, and the cell passage protocol was undertaken to determine the number of cells per square centimetre. The six cell counts were used to plot the cellular growth curve. For assessing the long-term growth, cells at day 6 were seeded again for two more passages.

### Colony-forming unit fibroblast (CFUF) assay

Five replicates were seeded in T25 flasks at 100 cells per square centimetre for 12 days with complete medium change every 3 days. On day 12, the culture medium was discarded, and flasks washed with PBS. Colonies were then fixed by incubating with 4% (w/v) paraformaldehyde solution (Fisher Scientific, UK) for 30 min at room temperature. After incubation, the flasks were rinsed with PBS and then stained with 1% (w/v) crystal violet (Sigma, UK) solution in methanol for 60 min. After incubation, the flasks were washed with excess distilled water then tapped on a towel and allowed to dry. Colonies formed of more than 25 cells were counted.

### Human BM-MSC differentiation

The assessment of the hBM-MSC differentiation potential was conducted via incubation with osteogenic and adipogenic differentiation media for 16 and 19 days, respectively. All media were prepared in-house, and complete medium change was performed every 3 days [17].

For osteogenic differentiation, cells were seeded at 5000 cells per square centimetre in complete growth media in 24-well plate for 24 h. After 24 h, the growth media were replaced with differentiation media consisting of 10% (v/v) FBS, 100 nM dexamethasone (Sigma, UK), 10 mM sodium β-glycerophosphate (Sigma, UK) and 0.05 mM ascorbic acid (Sigma, UK) in DMEM high glucose. For the osteogenic differentiation staining and quantification, an osteogenesis quantitation kit (Merck, UK) was used according to the manufacturer’s instruction. In brief, at day 16 in culture, the media were removed, and wells washed with PBS. Cells were fixed with 4% (w/v) paraformaldehyde for 15 min at room temperature then washed three times with distilled water. One millilitre/well Alizarin Red stain solution was added, and cells incubated for 20 min. After incubation, wells were washed four times with distilled water and imaged. To elute the dye, 400 μL of 10% acetic acid was added to each well and incubated for 30 min with shaking at 750 rpm. The monolayer was then scraped, and both the cells and the acetic acid were transferred to a 1.5-mL micro-centrifuge tube, vortexed and heated to 85 °C for 10 min. The tubes were then placed on ice for 5 min then centrifuged for 15 min at 17,000*g*; 150 μL of the supernatant was transferred to an opaque-walled, transparent-bottom 96-well plate and absorbance read at 405 nm using the Varioskan Flash spectral scanning multimode reader (Thermo Scientific, UK).

For adipogenic differentiation, cells were seeded at 10,000 cells per square centimetre in complete growth media in 12-well plate. After 24 h, the growth media was replaced with differentiation media which consisted of 10% (v/v) FBS, 1 mM dexamethasone, 0.5 mM IBMX (Sigma, UK) and 50 μM indomethacin (Sigma, UK) in DMEM high glucose. The staining and quantitation protocols were based on [18]. At day 19 in culture, the media was removed, and wells washed with PBS. Cells were fixed with 4% (w/v) paraformaldehyde for 15 min. Then, 0.2% (w/v) Oil Red O in 40% isopropanol was filtered and added to each well for 30 min. After that, wells were washed and imaged. To elute the dye, 1 mL of 100% isopropanol was added to each well and plates incubated for 10 min on a plate shaker at room temperature; 200 μL of eluate was transferred to a clear 96-well plate and absorbance read at 510 nm using the Varioskan Flash spectral scanning multimode reader (Thermo Scientific, UK).

Chondrogenic differentiation was conducted on fresh cells in order to complement the BM-MSC identity. Please refer to additional file 3.

### Statistical analyses

All data are presented as mean ± SD. The Shapiro-Wilk test and Levene’s test were used to check the normality of distribution and homogeneity of variance, respectively. For normally distributed data and when two time points were compared, independent *t* test was used to determine the significant differences between fresh and cryopreserved conditions for each cell line. When data was not normally distributed, Mann-Whitney *U* was used. When more than two time points were compared, one-way between-groups analysis of variance (ANOVA) was used as a parametric test and Kruskal-Wallis *H* was used as a non-parametric test. The results were deemed to be significant if *p* ≤ 0.05. To assess the correlation, the residuals of each variable were created and checked for normality and homogeneity of variances. Based on the results, Pearson’s product-moment correlation was used as a parametric test, and Spearman’s rank-order correlation was used as a non-parametric test. All statistical tests were conducted using the SPSS (IBM, USA, version 24) statistical software.

## Results

### The assessment of phenotypic marker expression

According to the ISCT, ‘≥ 95% of the MSC population must express CD105, CD73 and CD90, as measured by multiparameter flow cytometry. Additionally, these cells must lack expression (≤ 2% positive) of CD45, CD34, CD14 or CD11b, CD79a or CD19 and HLA class II’ [15]. Hence, the expression of surface markers was assessed at 0 h, 2 h, 4 h and 24 h post-thaw to determine an expression pattern and/or to detect any change in the level of expression. Cell surface marker expression in fresh cells (continuous culture) was considered the benchmark to which expression in cryopreserved cells was compared.

**Fig. 1:**
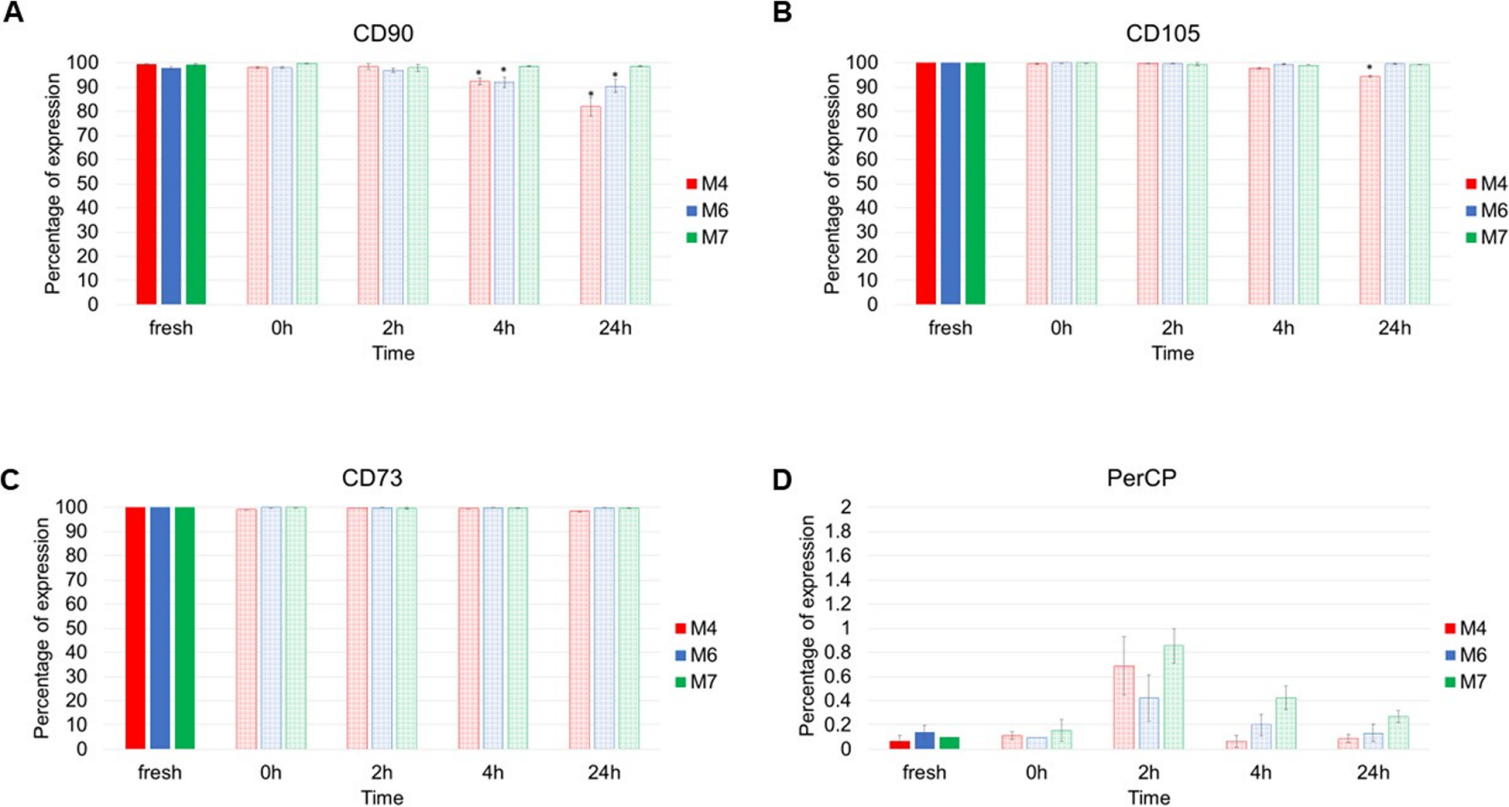
The expression of surface markers in fresh cells (solid histograms) and cryopreserved cells at 0 h, 2 h, 4 h and 24 h post-thaw (patterned histograms). **a** CD90, **b** CD105, **c** CD73 and **d** CD14, CD20, CD34, CD45 and HLA-DR are all linked to PerCP. Data is presented as the average of triplicates from three different experiments. Bars represent ± SD. Statistical tests carried only for expression level ≤ 95% for positive markers or ≥ 2% for negative markers. *Significant statistical difference compared to fresh at *p* ≤ 0.05

The expression of surface markers was assessed by flow cytometry using the MSC phenotyping kit (human) and according to the manufacturer’s instructions. Figure 1 shows the average percentage of the expression of each marker at each time point. The percentage of the expression of positive and negative markers in fresh cells across the three cell lines confirms the compliance with the ISCT criteria to define the cells used as MSCs. By examining the data from cryopreserved cells, the same pattern of expression is revealed across all of the markers at 0 h and 2 h post-thaw. However, there was a significant reduction below 95% in the expression of CD90 at 4 h (92.3% in M4 and 91.9% in M6) and 24 h (81.9% in M4 and 90.4% in M6) in two of the cell lines but not in the third (M7). The expression of CD105 and CD73 remained stable at 4 h and 24 h post-thaw except for a slight decrease below 95% in CD105 for the M4 cell line (94.3%) at 24 h post-thaw. The expression of negative markers increased at 2 h post-thaw but remained well below 2% across all of the time points for all of the cell lines.

There is a general agreement in the literature that cryopreservation does not affect marker expression in BM-MSCs [2]. The immediately and 2 h post-thaw data presented in this section is in line with this consensus. However, when marker expression was assessed at later time points post-thaw, a decrease below 95% in CD90 in two of the three cell lines and in CD105 in one cell line emerged.

Representative histograms for all data measured using flow cytometry were included in Additional file 1.

### The assessment of cell viability and apoptosis level

**Fig. 2:**
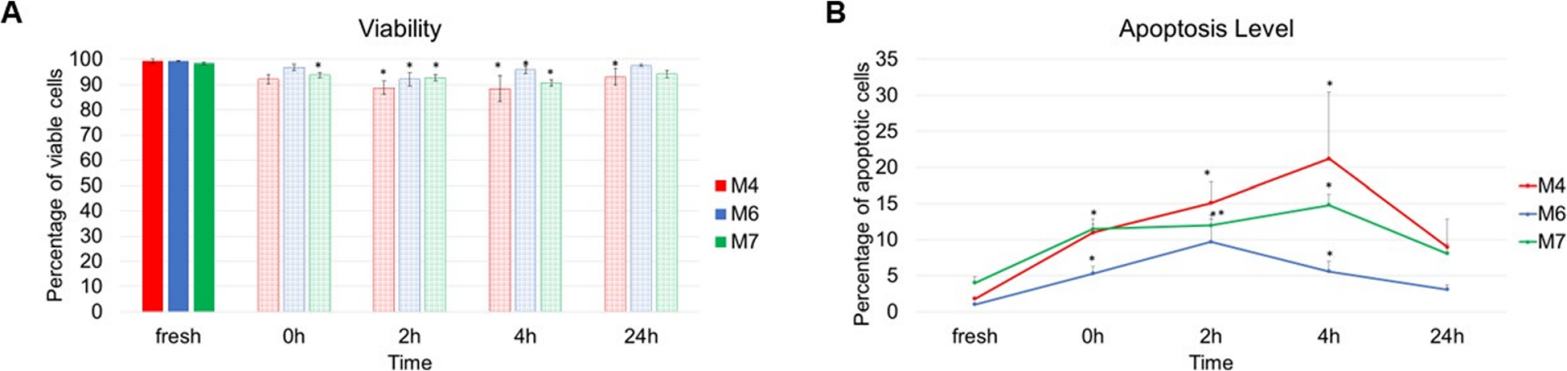
**a** Percentage of live cells in fresh samples (solid histograms) and cryopreserved samples at 0 h, 2 h, 4 h and 24 h post-thaw (patterned histograms). **b** Percentage of apoptotic cells in fresh samples and cryopreserved samples at 0 h, 2 h, 4 h and 24 h post-thaw. Data is presented as the average of triplicates from three different experiments. Bars represent ± SD. *Significant statistical difference compared to fresh at *p* ≤ 0.05

Cell viability remains the key criteria of a cryopreservation process and is certainly the most commonly used assay after BM-MSC thawing. Measuring cell viability and apoptosis at different time points post-thaw is important to revealing the cell response to freezing and thawing, not only immediately but also after several hours post-thaw as delayed onset apoptosis may be triggered. Understanding the complete time frame is vital especially if cells are intended for intravenous injection upon thawing or after few hours due to delays in operation theatre (either foreseen or unforeseen procedural timings). The data on viability and the level of apoptotic cells in fresh cells and at 0 h, 2 h, 4 h and 24 h post-thaw for the three cell lines are presented in Fig. 2.

Viability across the three cell lines for fresh samples and at all time points after thawing shows the same pattern. As measured by flow cytometry using 7AAD, the viability in fresh samples was well above the benchmark for clinical application which is ≥ 90% [19] (99.1% for M4, 99.2% for M6 and 98.4% for M7). Immediately post-thaw, viability started to decrease but was significantly lower than fresh for one cell line only (93.8% for M7). At 2 h and 4 h post-thaw, all three cell lines had significantly lower viability compared to fresh at *p* ≤ 0.05 (88.8% for M4, 92.1% for M6 and 92.8% for M7 at 2 h; 88.4% for M4, 95.8% for M6 and 90.7% for M7 at 4 h). At 24 h post-thaw, cell viability levels showed recovery for the three cell lines (93.1% for M4, 97.6% for M6 and 94.3% for M7) (Fig. 2a).

Notably, the level of apoptosis closely corresponds to the viability levels as measured using Annexin V staining. In fact, Annexin V has a high affinity to the cell membrane component phosphatidylserine (PS). In normal cells, PS resides on the intracellular side of the cell membrane. When apoptosis is initiated, PS is translocated to the outer surface of the membrane, and this is considered an early sign of apoptosis [20, 21]. In fresh samples, viability measures were the highest. Equivalently, the levels of apoptotic cells in fresh samples were the lowest (1.8% for M4, 1% for M6 and 4% for M7). At 0 h post-thaw, the apoptosis level started to increase for the three cell lines (11% for M4, 5.3% for M6 and 11.5% for M7) and continued to increase at 2 h and 4 h post-thaw except for the M6 cell line which showed some recovery at 4 h (15.1% for M4, 9.7% for M6 and 12% for M7 at 2 h; 21.2% for M4, 5.6% for M6, 14.8% for M7 at 4 h). At 24 h post-thaw, the levels of apoptosis reduced for the three cell lines and were not significantly different from fresh samples (9% for M4, 3.1% for M6, 8.1% for M7) (Fig. 2b).

Apoptosis is the most common cell death mode associated with cryopreservation. Very few studies assessed apoptosis as a result of freezing and thawing in BM-MSCs [2] (p. 23). Hence, it is important to collect this data in order to elucidate BM-MSCs’ tolerance to freezing and its impact on cell viability up to 24 h post-thaw to enable the elucidation of an effective therapeutic window. The viability and apoptosis data presented above were tested for statistical correlation. For the three cell lines, a strong and significant negative relationship exists between viability and apoptosis (rho = − 0.868 for M4, − 0.954 for M6 and − 0.920 for M7). This means that as the level of viability decreases across a cell line, the level of apoptosis increases proportionally. The coefficient of determination suggests that a change in viability could explain about 75%, 91% and 85% of the variance in M4, M6 and M7 apoptosis level, respectively (*r*^2^ = 0.75, 0.91 and 0.85, respectively).

### The assessment of cell metabolic activity

**Fig. 3:**
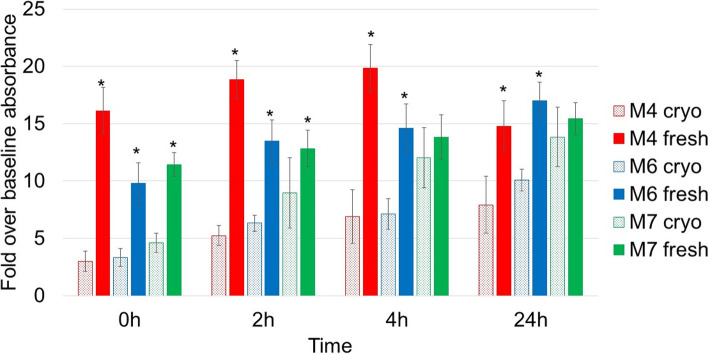
Level of metabolic activity in fresh samples (solid histograms) and cryopreserved samples at 0 h, 2 h, 4 h and 24 h post-thaw (patterned histograms). Data is presented as the average of triplicates from three different experiments. Bars represent ± SD. *Significant statistical difference between cryopreserved and fresh samples at each time point at *p* ≤ 0.05

Cells’ metabolic activity was measured using the colourimetric assay WST-1. The level of metabolic activity was assessed in fresh cells at 0 h, 2 h, 4 h and 24 h post-seeding and in cryopreserved cells at 0 h, 2 h, 4 h and 24 h post-thaw, and data is presented in Fig. 3. For the three cell lines and at all the time points, cell activity in cryopreserved cells was lower than fresh which strongly indicates that the freeze-thawing cycle slows down cells’ metabolism even at 24 h post-thaw. Of note, the M7 cell line showed a faster recovery compared to M4 and M6 with the cell activity levels not significantly different from fresh at 4 h and 24 h post-thaw.

### The assessment of adhesion potential

**Fig. 4:**
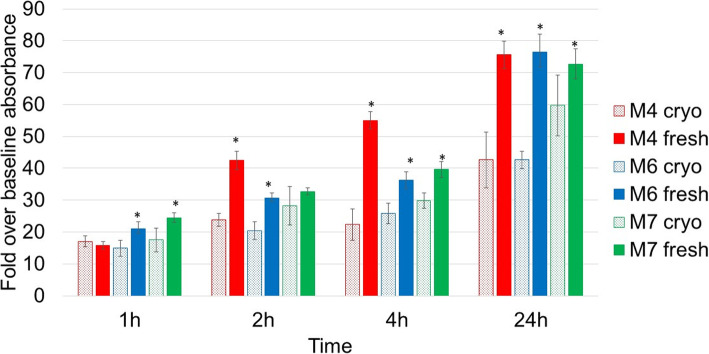
Adhesion potential in fresh samples at 1 h, 2 h, 4 h and 24 h post-seeding (solid histograms) and cryopreserved samples at 1 h, 2 h, 4 h and 24 h post-thaw (patterned histograms). Data is presented as the average of triplicates from three different experiments. Bars represent ± SD. *Significant statistical difference between cryopreserved and fresh samples at each time point at *p* ≤ 0.05

The ability to adhere to a substrate is an important characteristic for isolating and defining MSCs and is vital for both in vitro and in vivo cell functioning. Therefore, cell adhesion was assessed in fresh cells at 1 h, 2 h, 4 h and 24 h post-seeding and in cryopreserved cells at 1 h, 2 h, 4 h and 24 h post-thaw. In Fig. 4, it is evident that cryopreservation significantly reduced cells’ ability to adhere at almost all time points. As expected, with longer incubation time, the level of adherence increased but remained significantly lower in cryopreserved cells compared to fresh.

### The assessment of cell growth/proliferation

**Fig. 5:**
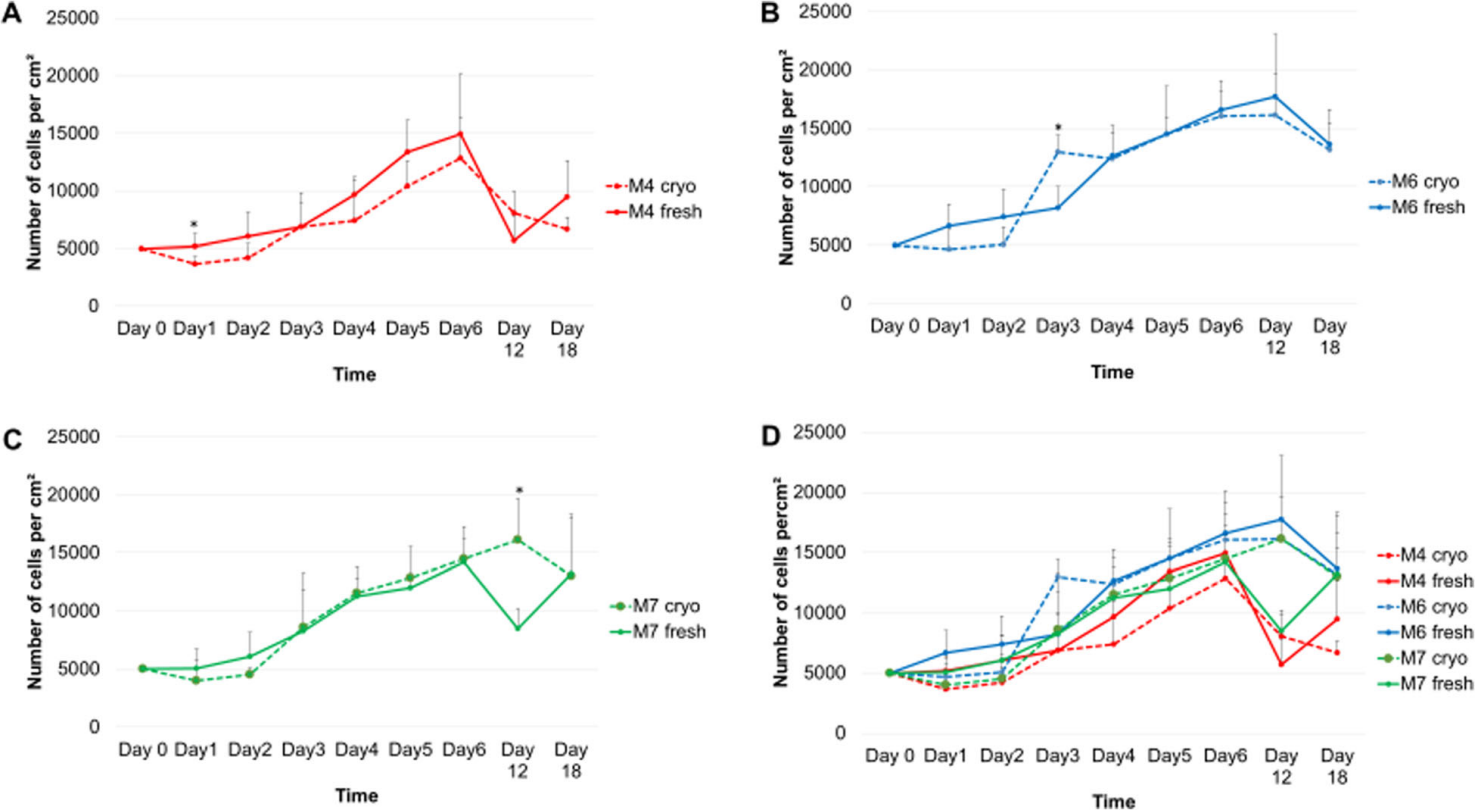
Growth curves of fresh and cryopreserved cell lines: **a** M4 cell line, **b** M6 cell line, **c** M7 cell line and **d** the same growth curves for the three cell lines combined in order to show the similarity in the growth pattern. Data is presented as the average of duplicates from three different experiments. Bars represent + SD. *Significant statistical difference between fresh and cryopreserved at *p* ≤ 0.05

In order to determine the growth of fresh and cryopreserved cells of each cell line, six T25 flasks at a density of 5000 cells per square centimetre were seeded. At days 1 to 6 each, one flask was removed from the incubator and the cell passage protocol was undertaken to determine the number of cells per square centimetre. The six cell counts were used to plot the growth curve of each cell line (Fig. 5). For assessing the long-term growth, cells at day 6 were seeded again for two more passages (Fig. 5). The three cell lines showed very similar growth dynamics with a significantly statistical difference between fresh and cryopreserved only at day 1 for M4 and at day 3 for M6 (Fig. 5a, b respectively). In the following two passages, fresh and cryopreserved cells continue to exhibit similar proliferative pattern, and a significant statistical difference is only seen for the M7 cell line at day 12 in culture post-thaw (Fig. 5).

### The assessment of colony-forming unit fibroblast (CFUF) ability

**Fig. 6:**
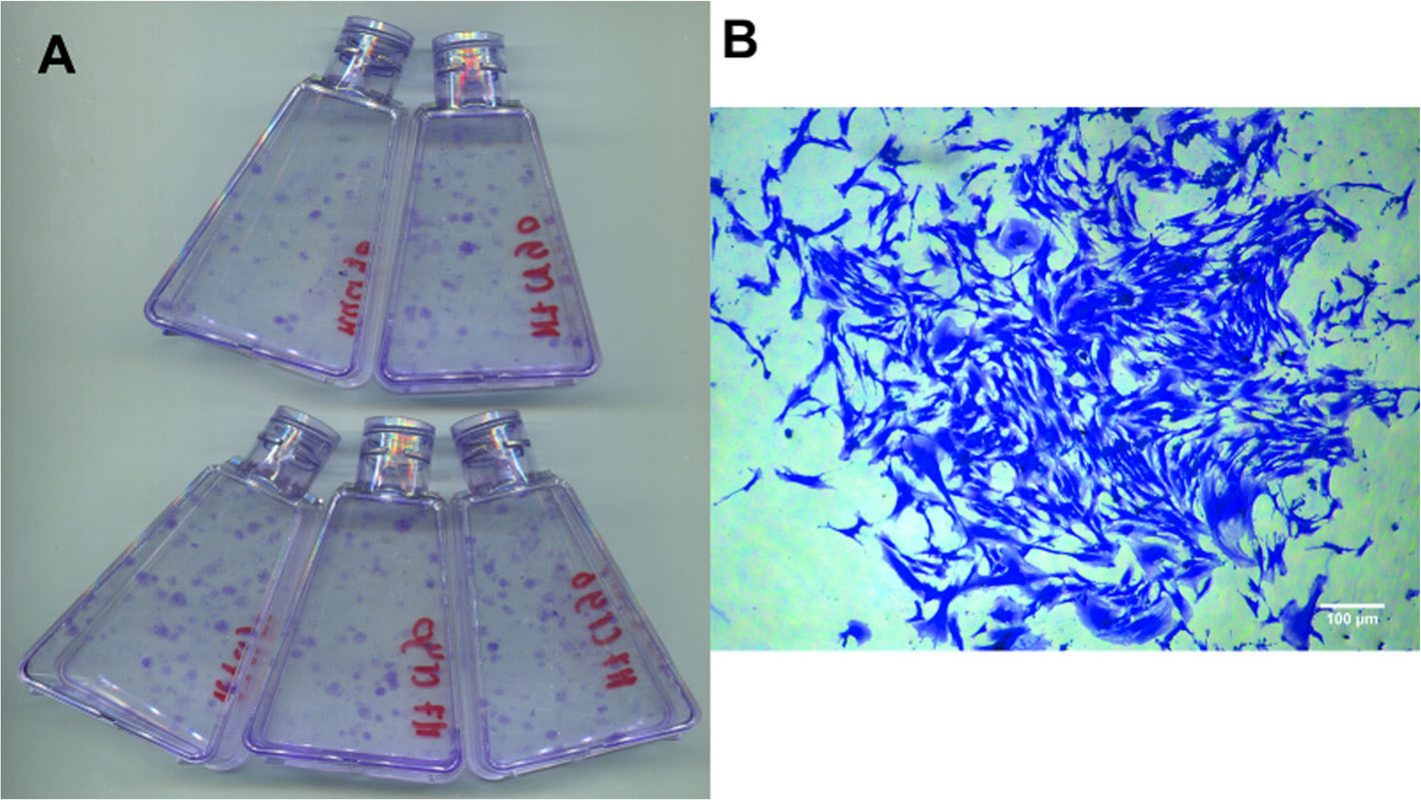
**a** A representative scan of flasks prepared for colony counting. **b** A representative microscopic image of a stained colony, scale bar represents 100 μm

**Fig. 7:**
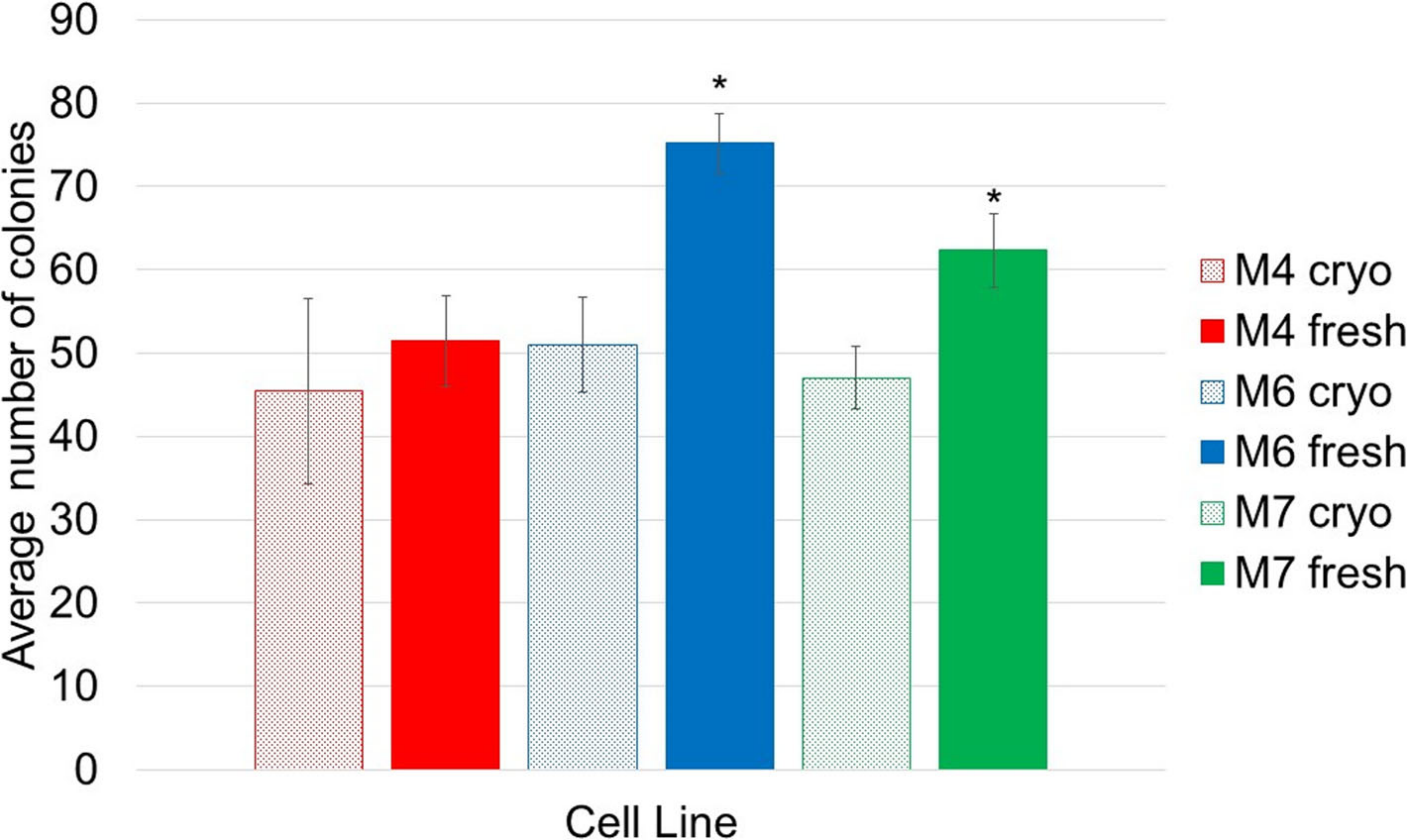
Colony-forming unit fibroblast (CFUF) in fresh cells (solid histograms) and cryopreserved cells (patterned histograms). Data is presented as the average number of colonies in five replicates from three different experiments. Bars represent ± SD. *Significant statistical difference between fresh and cryopreserved at *p* ≤ 0.05

The colony-forming unit ability of hBM-MSCs before and after cryopreservation was determined by seeding the cells at low density in normal growth conditions for 12 days. After 12 days, the colonies formed were fixed and counted (Fig. 6). Data for the three cell lines is presented in Fig. 7. No significant difference in the number of colonies was found for the M4 cell line. However, for the M6 and M7 cell lines, fresh cells from continuous culture formed more colonies than cryopreserved cells (75.2 ± 3.6 vs 51 ± 5.7 and 62.3 ± 4.5 vs 47.1 ± 3.7, respectively). This data agrees with the literature where [22] found that cryopreserved porcine BM-MSCs showed a significantly lower number of colonies compared to fresh while [23] reported that cryopreservation did not affect the colony-forming ability of horse BM-MSCs. It can be suggested here that the effect of cryopreservation on BM-MSCs CFUF ability may be mediated by cell source and/or cell donor.

### The assessment of cell differentiation ability

The ability of MSCs to differentiate is one of the ISCT characteristics to define these cells. To confirm compliance with these criteria, all three cell lines were differentiated into adipocytes and osteoblasts. Induction of differentiation into these lineages was achieved by incubating the cells in at-home made differentiation media for a various number of days (19 days for adipogenesis and 16 days for osteogenesis). After incubation, cells were washed, fixed and stained with Oil Red O and Alizarin Red, respectively. Next, Oil Red O and Alizarin Red staining were quantified in fresh and cryopreserved cells.

The microscopic examination of cell morphology during incubation of MSCs in differentiation media can reveal changes in the cell shapes and sizes which indicate turning into more specialised cell types while control culture for both adipogenesis and osteogenesis retained their fibroblastic appearance and no structural change occurred. Light microscopy images of positively stained cells and control cells which stained negative were taken. Positively stained fresh and cryopreserved cells show Oil Red O staining of lipid droplet and Alizarin Red staining of calcium mineralisation and nodule formation (which is a characteristic of osteogenic differentiation) (Figures S9 to S15 in Additional file 2).

**Fig. 8:**
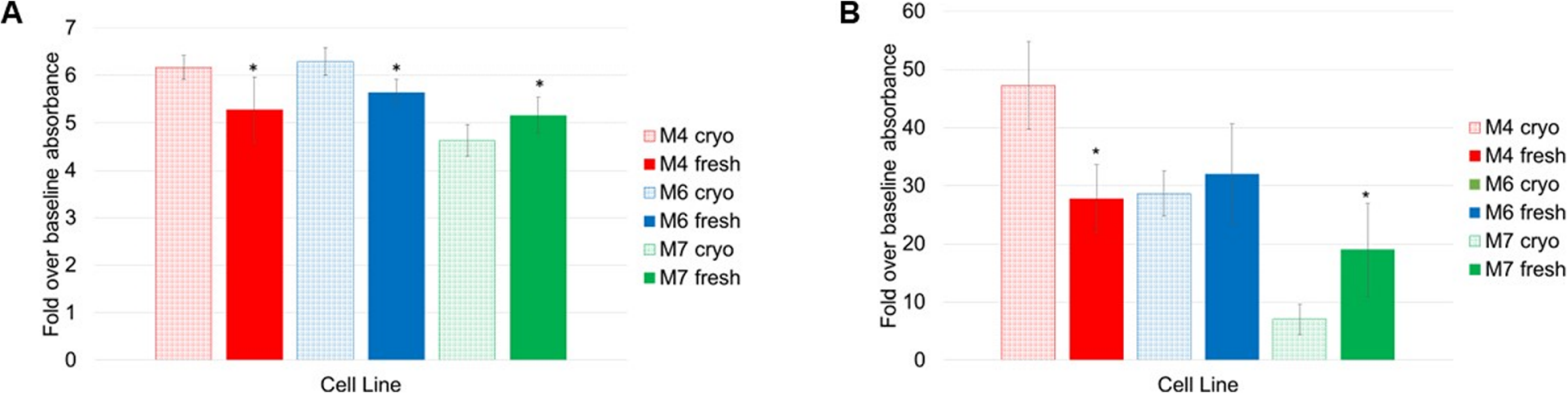
**a** Quantification of adipogenic differentiation of fresh cells (solid histograms) and cryopreserved cells (patterned histograms). **b** Quantification of osteogenic differentiation of fresh cells (solid histograms) and cryopreserved cells (patterned histograms). Data is presented as the average of triplicates from three different experiments. Bars represent ± SD. *Significant statistical difference between fresh and cryopreserved at *p* ≤ 0.05

By examining the adipogenesis and osteogenesis images, it can be confirmed that the three cell lines fresh and cryopreserved differentiated into these two lineages. However, this qualitative assessment gives little to no evidence on the difference in the differentiation potential between fresh and cryopreserved cells. It is impossible to qualitatively judge if and how cryopreservation affected cells’ differentiation level. Therefore, to more accurately evaluate the impact of cryopreservation on the adipogenic and osteogenic differentiation of the three cell lines, the staining was followed by quantitative assays. For the three cell lines, a statistically significant difference between fresh and cryopreserved adipogenic potential was found. For M4 and M6 cell lines, cryopreserved cells have a higher differentiation level compared to fresh while for M7 cell line, cryopreserved cells have a lower differentiation level (Fig. 8a). Likewise, cryopreservation influenced the three cell lines’ osteogenic potential differently. Cryopreserved M4 cells have higher a differentiation level than fresh, no significant difference is observed for the M6 cells and cryopreserved M7 cells have lower differentiation level (Fig. 8b). The data presented here point in the direction that the effect of cryopreservation on the differentiation ability of hBM-MSCs is cell line-dependent.

## Discussion

The known effects of cryopreservation on BM-MSCs in terms of cellular attributes and function were discussed in detail in a recent review [2]. From the systematic analysis performed, it was concluded that cryopreservation has an impact on viability and apoptosis, cellular attachment, immunomodulation and metabolism. However, no common significant impact caused by cryopreservation has been reported in proliferation, morphology, differentiation or immunophenotyping [2]. Across the studies in the literature, there was no general freezing protocol followed, there was a lot of variability in the freezing media used, the assessed criteria were not always in common and the methods of assessment also varied. In addition, assays—within a study and across multiple studies—were often performed on cells from several different passage numbers. All these factors made it hard to compare and draw firm and reliable conclusions regarding the impact of cryopreservation on BM-MSCs.

This study aims to provide new systematic and coherent evidence on the impact of cryopreservation on hBM-MSCs in the first 24 h post-thaw and beyond. Through quantitative assays performed pre- and post-cryopreservation, viability, expression of surface markers, apoptosis, adhesion potential, metabolic activity, proliferation, colony-forming ability and differentiation potentials were assessed. All the assays were performed on hBM-MSCs derived from three different donors, so donor variability could be accounted for. All the cells used in this study (fresh and cryopreserved) were from the same passage (P4) to ensure consistency across the assays and the various time points. The assays chosen were quantitative and assess important cell functions which reflect cells’ health and fitness to be used as a therapy. Numerous studies in the literature have addressed the impact of cryopreservation on hBM-MSCs, but none of them followed a similar framework to understand the dynamics of changes which occur in the first 24 h after thawing. In particular, across the literature, metabolic activity, adhesion potential and apoptosis level were considered in only 3, 2 and 7 studies using BM-MSCs from a human source, respectively [2].

In this study, viability and apoptosis levels were assessed using flow cytometry. Shrinkage was observed in cryopreserved cells and was closely associated with a higher level of apoptosis. In fact, a fall in viability and an increase in apoptotic rate were observed immediately post-thaw. Viability continued to significantly drop, and apoptosis rate became significantly higher at 2 h and 4 h post-thaw (Figs. 2). In fact, it is known that the exposure to DMSO, in addition to the physical and molecular changes caused by freezing and thawing, compromises cell viability [24]. The results presented herein are in accordance with the literature and can be further explained by the phenomenon known as cryopreservation-induced delayed-onset cell death [25]; the highest change in viability and apoptosis levels in this study were observed at 4 h post-thaw. At 24 h post-thaw, no significant difference (except for M4 viability) was observed between fresh and cryopreserved samples, and most importantly, viability levels start to increase, and apoptosis rate starts to decrease. Two possible explanations to this observed pattern can be suggested, the first is culture re-population and the second is reversible apoptosis. It was shown that early apoptotic cells can recover and revert to proliferative cells if the apoptotic provocation is removed [26]. The thawing protocol in this experiment was rapid and was followed by a washing step aimed at removing any remnant DMSO. Cells seeded for the 24 h time point were incubated in complete media in Nunc™ cell culture-treated flasks with filter caps (Fisher Scientific) in a humidified incubator. These represent ideal conditions for the MSCs to recover from freezing. However, this in vitro setup may not reflect the in vivo environment of a therapy injection site such as a diseased or injured tissue where cells would continue to be exposed to various stimuli including apoptotic ones.

In addition to testing viability and apoptosis, surface marker expression was also analysed using flow cytometry (Fig. 1). This is important to ensure that cryopreserved cells maintained their mesenchymal identity during the 24-h time course. The data showed that the expression of surface markers, both positive and negative, are mostly stable after cryopreservation except for CD90, one of the main markers for defining MSC. In a recent editorial article, CD90, also known as Thy-1, was described as a microenvironment sensor [27] and more than a marker for mesenchymal stromal cells [28]. In fact, the expression of CD90 on MSC was mainly associated with fate decision, namely promotion of osteogenesis accompanied by inhibition of adipogenesis [28, 29]. However, the knockdown of CD90 expression in MSC from various sources has been shown to promote both osteogenic and adipogenic differentiation and reduce the cells’ stemness properties [30]. Here, for the first time, we provide evidence that a delayed effect of cryopreservation is a decrease in the expression of CD90 which can be translated as the cells’ readiness to commit to a differentiated phenotype. It is worth noting here that this CD90 decrease was only observed in two of the cell lines used (M4 and M6 but not in M7). This reduction in CD90 expression aligns well with the differentiation data where the cryopreserved M4 cell line showed higher osteogenic and adipogenic differentiation levels, and the M6 cell line showed higher adipogenic differentiation and unchanged osteogenic differentiation levels (Fig. 8a, b). This may indicate that this cryopreservation effect is donor-dependent.

MSC differentiation was for some time considered the most attractive characteristic of MSCs and its main regeneration mechanism. However, recently, this theory has been challenged by MSC paracrine activity. Despite this, MSC differentiation remains a very important assay for defining this type of cells [15] and for proving their multilineage potential. MSC differentiation assays are very often employed as qualitative with osteogenic differentiation being quantified the most [2]. Evaluating the differentiation after cryopreservation is prevalent with a general conclusion that freezing and thawing do not affect this MSC ability [2]. It is well known that variabilities govern many facets of MSCs including, but not limited to, tissue source, donor-to-donor variability, isolation procedures and heterogeneity of the composition of the isolated sample [31–35]. Consequently, these variabilities affect MSC function capabilities and represent a major and an ongoing challenge for MSC manufacturing. In this specific investigation, the variable effects of cryopreservation on the differentiation of the three cell lines could lend itself to donor variability with a note that cells used in this project were isolated in the same lab and then were processed by one operator using the same protocols throughout the entire investigation.

In addition to CD90, cell adhesion and physical cues have also been associated with MSC differentiation [36]. Specifically, cell adhesion to the extracellular matrix has been shown to promote osteogenic differentiation through spreading (a large number of focal adhesions) and tension while averting adipogenic differentiation, promoted by the formation of few but strong focal adhesions [36–38]. Moreover, MSC ability to adhere to the endothelium is essential to ‘MSC homing’, a crucial multistep process that allows therapeutic cells to reach their target area [39, 40]. Karp and Teo [39] concluded that in vitro-cultured MSC lack the expression of certain surface adhesion molecules which lowers their homing ability, and here, our data provides further evidence that cultured cryopreserved cells have a significantly lower adhesion ability up to 24 h post-thaw when compared to fresh (Fig. 4) and this is in agreement with the literature [2]. Therefore, it can be suggested that cryopreservation diminishes MSC homing ability and influences the cell’s fate and differentiation. Chinnadurai et al. [41] referred MSC post-thaw low adherence capacity to disruption of F actin polymerisation rather than shedding of surface adhesion molecules. Further studies in this area could elucidate the adhesion mechanisms disrupted by cryopreservation and inform strategies to avoid this effect.

To further investigate the effect of cryopreservation on MSC, the effect on cells’ metabolic activity was analysed. Data presented in Fig. 3 clearly shows that cryopreservation impacted cellular metabolism by slowing it down across the 24-h period. With a note that the M7 cell line’s improved metabolism reflects cells’ ability to recover from the freezing journey and, again, clearly indicates the biological variation among the three hBM-MSC cell lines. This variation may be linked to the donors’ age and ethnicity (Table 1).

The proliferation of hBM-MSCs is a particularly a vital characteristic for the scale-up of allogeneic therapies where a certain number of doses must be produced within a specific time frame following a standardised protocol. MSC proliferation has been widely studied in terms of starting material (donors’ age and health), culture systems and conditions as well as media and its supplementation. It is clearly important to assess the impact downstream processes, namely cryopreservation, have on the proliferation potential of hBM-MSCs. It is shown in Fig. 5 that the proliferation rate of post-cryopreservation MSCs is not statistically different from the continuous-culture control cells, suggesting a possible complete recovery from the cold journey. Nevertheless, from a closer look at the growth curves of the three lines, it can be noted that cryopreserved cells grew at a slower rate than fresh for the first 2 days then matched the level of growth of fresh cells from day 3 and beyond. The lower cell numbers in the first 2 days can be explained by the lower adhesion capability of cryopreserved cells (discussed above). This means that the same proliferation rates at days 6, 12 and 18 (Fig. 5) were observed for each cell line even though cryopreserved cultures started off with less cells. This observation can be linked to the possible process of ‘cell selection’ described in Ginis et al. [42] which showed that BM-MSC post-thaw proliferation potential was higher than that at pre-freeze. The authors justified their results as a selection of stronger cells after cryopreservation and suggested that their results should alarm a scientific community. This same theory has also been mentioned by Baust et al. [43] who stated, ‘unstudied but of concern is the potential for the preservation process to select for increased resistance to preservation stresses’. In this section, we provide what could be a further evidence on this ‘cellular natural selection’.

Another measure of MSC proliferation, quality and stemness is their ability to form colonies (CFUF). CFUF was described as one of the most eminent characteristics of MSCs [44]. When plated at a low density, MSCs form distinctive colonies and it is assumed that each colony originates from a single progenitor cell (in the case of hMSCs). Hence, this assay is regarded as suitable for estimating the percentage of early progenitors in MSC samples and for evaluating the biological activity of discrete cells [45]. In other words, it can be considered as another measure of MSCs potency. CFUF was shown to decline during successive culture and passaging [46]. Data on how cryopreservation affects hBM-MSCs CFUF is scarce with only three studies showing mixed results [22, 23, 47]. Figure 7 shows a comparison of the colony-forming abilities of M4, M6 and M7 before freezing and after thawing. Despite the similarities in the growth kinetics among the three cell lines, cryopreserved cultures formed less colonies compared to fresh. Of note here is that the difference was statistically significant for only two (M6 and M7) out of the three cell lines assessed. This result is significant if cryopreserved cells are to be used as starting material or as an end-product in MSC manufacturing. On the one hand, when developing large-scale manufacturing processes which are likely to require lot sizes in excess of a trillion cells [48], losing progenitor cells at the beginning of the process can intensely affect the product yield. This in turn would represent a burden on the financial resources. On the other hand, if the end product is to be characterised pre- and post-cryopreservation, the discrepancy in the CFUF (and of the many characteristics presented so far) risks failing the quality tests (in specific potency) and meeting release criteria.

## Conclusion

Cryopreservation is vital to make MSC therapies widely available and affordable. It is the way forward for stocking and delivering MSCs in the quality and quantity needed. The demand on MSC therapies is continuously , and the high hopes on product efficacy are being increasingly supported by more scientific evidence as research in this area is dramatically growing. Manufacturing autologous and allogeneic hBM-MSC therapies requires rigorous characterisation of the input material and of the end-product. Given that cryopreservation is most certainly an integral part of the manufacturing and delivery processes, its impact on the cells and any introduction of variation in the process must be assessed and addressed.

From the quantitative assessment of the impact of cryopreservation on hBM-MSCs presented in this study, it can be concluded that cryopreservation has no effect on the cells’ proliferation ability but reduces cell viability, metabolic activity, adhesion potential and the colony-forming ability. In addition, the effects on the differentiation ability are cell line-dependent. It is important to note that this study shows that a 24-h recovery period can allow hBM-MSCs to recover but not fully. With such differences between fresh and cryopreserved hBM-MSCs, the variabilities that cryopreservation can introduce to the product and process development are challenging and may have significant financial implications. Effective strategies must be implemented in order to eliminate or reduce the biological divergence of the product pre- and post-cryopreservation. This will certainly yield higher-quality and more consistent hBM-MSC products. All experiments in this work were conducted using a cryopreservation medium composed of 90% (v/v) FBS and 10% (v/v) DMSO which is not acceptable for human infusion. The transfer of this work to other cryopreservation media with lower concentrations of FBS and DMSO and to FBS and DMSO free alternative cryoprotectants is necessary. In addition, it is of high importance to compare this work’s results with those of commercially available cryopreservation media such as CryoStor CS2, CS5 and CS10 which modulate the cellular biochemical response to the cryopreservation process.

## Supplementary Information

**Additional file 1: Figures S1-S8.** Representative histograms for immunophenotyping, viability and apoptosis measures for the three lines (fresh and at 0h, 2h, 4h and 24h post-thaw). **Figure S1.** Immunophenotyping of fresh cells: Representative histograms of expression of CD90, CD105, CD73 and CD14, CD20, CD34, CD45 and HLA-DR all linked to PerCP. Black peaks represent isotype controls in all histograms. The first row of histograms is for M4 (red), the second for M6 (blue) and the third for M7 (green). All measurements were done in triplicates from three independent experiments based on at least 100,000 events. **Figure S2.** Immunophenotyping of cryopreserved cells at 0 h post-thaw: Representative histograms of expression of CD90, CD105, CD73 and CD14, CD20, CD34, CD45 and HLA-DR all linked to PerCP. Black peaks represent isotype controls in all histograms. The first row of histograms is for M4 (red), the second for M6 (blue) and the third for M7 (green). All measurements were done in triplicates from three independent experiments based on at least 100,000 events. **Figure S3.** Immunophenotyping of cryopreserved cells at 2 h post-thaw: Representative histograms of expression of CD90, CD105, CD73 and CD14, CD20, CD34, CD45 and HLA-DR all linked to PerCP. Black peaks represent isotype controls in all histograms. The first row of histograms is for M4 (red), the second for M6 (blue) and the third for M7 (green). All measurements were done in triplicates from three independent experiments based on at least 100,000 events. **Figure S4.** Immunophenotyping of cryopreserved cells at 4 h post-thaw: Representative histograms of expression of CD90, CD105, CD73 and CD14, CD20, CD34, CD45 and HLA-DR all linked to PerCP. Black peaks represent isotype controls in all histograms. The first row of histograms is for M4 (red), the second for M6 (blue) and the third for M7 (green). All measurements were done in triplicates from three independent experiments based on at least 100,000 events. **Figure S5.** Immunophenotyping of cryopreserved cells at 24 h post-thaw: Representative histograms of expression of CD90, CD105, CD73 and CD14, CD20, CD34, CD45 and HLA-DR all linked to PerCP. Black peaks represent isotype controls in all histograms. The first row of histograms is for M4 (red), the second for M6 (blue) and the third for M7 (green). All measurements were done in triplicates from three independent experiments based on at least 100,000 events. **Figure S6.** Apoptosis and viability measures of the M4 cell line: Representative overlay histograms of blank samples (black) and M4 samples stained with Annexin V and 7AAD (red) at different time points. All measurements were done in triplicates from three independent experiments based on at least 100,000 events. **Figure S7.** Apoptosis and viability measures of the M6 cell line: Representative overlay histograms of blank samples (black) and M6 samples stained with Annexin V and 7AAD (red) at different time points. All measurements were done in triplicates from three independent experiments based on at least 100,000 events. **Figure S8.** Apoptosis and viability measures of the M7 cell line: Representative overlay histograms of blank samples (black) and M7 samples stained with Annexin V and 7AAD (green) at different time points. All measurements were done in triplicates from three independent experiments based on at least 100,000 events.

**Additional file 2: Figures S9-S15.** fresh and cryopreserved cells stained positive for Oil Red O staining and Alizarin Red staining after incubation with adipogenic and osteogenic differentiation media. **Figure S9.** Oil Red O staining of fresh and cryopreserved M4 cells following 19 days of incubation in adipogenic differentiation media: Light microscopy representative images: (A) M4 fresh control well, (B) M4 fresh differentiation well, (C) M4 cryopreserved control well, (D) M4 cryopreserved differentiation well. Scale bars represent 100μm. **Figure S10.** Oil Red O staining of fresh and cryopreserved M6 cells following 19 days of incubation in adipogenic differentiation media: Light microscopy representative images: (A) M6 fresh control well, (B) M6 fresh differentiation well, (C) M6 cryopreserved control well, (D) M6 cryopreserved differentiation well. Scale bars represent 100μm. **Figure S11.** Oil Red O staining of fresh and cryopreserved M7 cells following 19 days of incubation in adipoegnic differentiation media: Light microscopy representative images: (A) M7 fresh control well, (B) M7 fresh differentiation well, (C) M7 cryopreserved control well, (D) M7 cryopreserved differentiation well. Scale bars represent 100μm. **Figure S12.** Alizarin Red staining of fresh and cryopreserved M4 cells following 16 days of incubation in osteogenic differentiation media: Light microscopy representative images: (A) M4 fresh control well, (B) M4 fresh differentiation well, (C) M4 cryopreserved control well, (D) M4 cryopreserved differentiation well. Scale bars represent 100μm. **Figure S13.** Alizarin Red staining of fresh and cryopreserved M6 cells following 16 days of incubation with osteogenic differentiation media: Light microscopy representative images: (A) M6 fresh control well, (B) M6 fresh differentiation well, (C) M6 cryopreserved control well, (D) M6 cryopreserved differentiation well. Scale bars represent 100μm. **Figure S14.** Alizarin Red staining of fresh and cryopreserved M7 cells following 16 days of incubation in osteogenic differentiation media: Light microscopy representative images: (A) M7 fresh control well, (B) M7 fresh differentiation well, (C) M7 cryopreserved control well, (D) M7 cryopreserved differentiation well. Scale bars represent 100μm.**Figure S15.** (A) Adipogenic differentiation: Representative zoomed image to clearly show the formation of lipid droplets and (B) Osteogenic differentiation: Representative zoomed image to clearly show the formation of a mineralized nodule.

**Additional file 3. **For chondrogenic differentiation, cells were centrifuged, and supernatant completely removed to obtain a cell pellet. 3x5μL droplets of concentrated cells were added to 12-well plate. The generated micro-masses were incubated for about an hour in a humidified incubator to allow attachment. After incubation, 1 mL/well of differentiation media was added. The differentiation media consisted of 100 nM dexamethasone, 10% ITS-Premix (Sigma, UK), 1 μg/mL ascorbic acid, 1% sodium pyruvate (Sigma, UK) and 10 ng/mL Human TGFβ1 (R&D systems, UK) in DMEM high glucose. At day 21 in culture, cells were fixed by incubating with paraformaldheyde then stained with filtered (1%w/v) Alcian Blue (Sigma, UK) in Hydrochloric acid. After one-hour incubation with the stain, wells were washed twice with distilled water and then imaged (Figure S16). **Figure S16.** Alcian Blue staining of fresh cells following 21 days of incubation with chondrogenic differentiation media: Representative images: (A) M4 cell line, (B) M6 cell line and (C) M7 cell line. Scale bars represent 100 μm.

## Data Availability

The datasets used and/or analysed during the current study are available from the corresponding author on reasonable request.

## Abbreviations

BM-MSCs: Bone marrow-derived mesenchymal stem cells
CFUF: Colony-forming unit fibroblast
DMEM: Dulbecco’s medium Eagle’s media
DMSO: Dimethylsulfoxide
FBS: Foetal bovine serum
hBM-MSCs: Human bone marrow-derived mesenchymal stem cells
hMSC(s): Human mesenchymal stem cell(s)
ISCT: International Society for Cellular Therapy
LN_2_: Liquid nitrogen
MSC(s): Mesenchymal stem cell(s)
P: Passage
PBS: Phosphate-buffered saline
PS: Phosphatidylserine
