# Supplementary figures and images for: Quantitative assessment of the impact of cryopreservation on human bone marrow-derived mesenchymal stem cells: up to 24 h post-thaw and beyond
Source: Stem Cell Res Ther. 2020 Dec 14;11:540. doi: 10.1186/s13287-020-02054-2 (PMC7734731; doi:10.1186/s13287-020-02054-2)

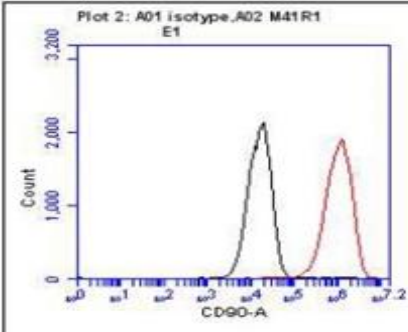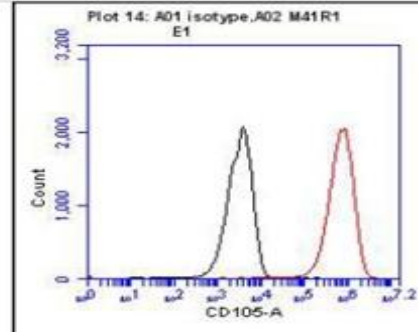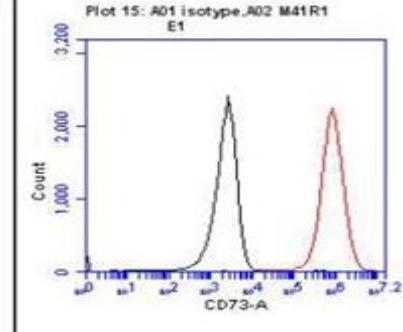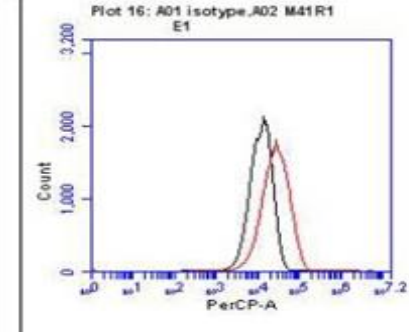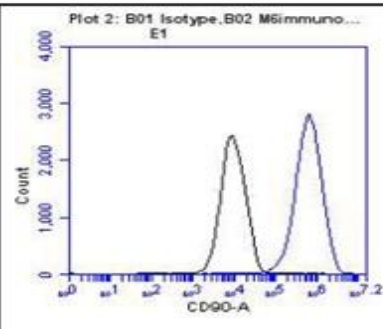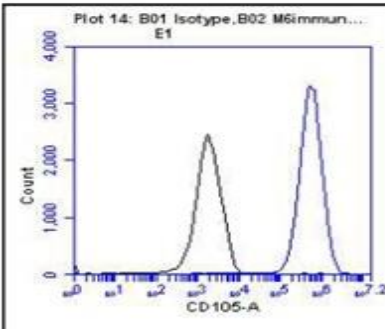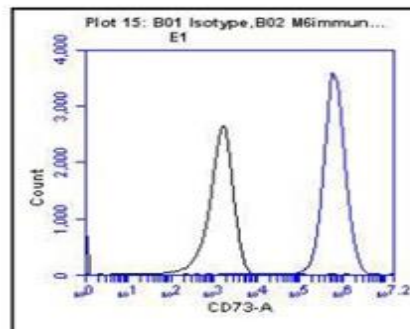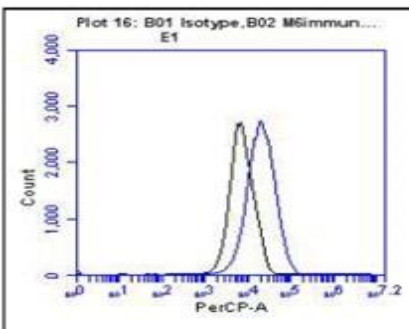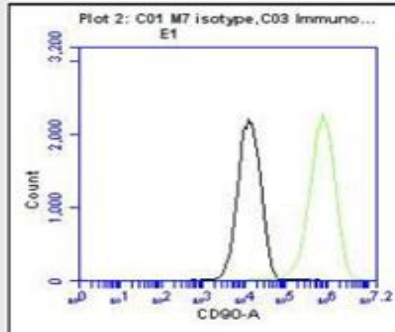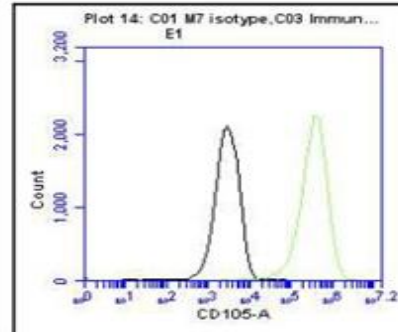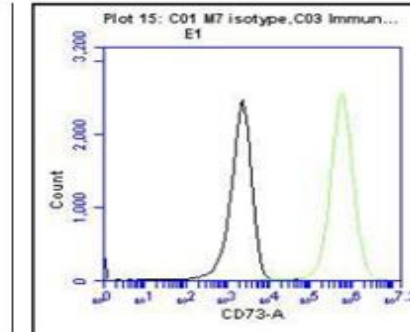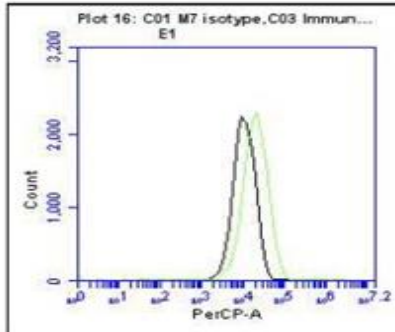

Supplement: Supplementary file 1 — Additional file 1: Figures S1-S8. Representative histograms for immunophenotyping, viability and apoptosis measures for the three lines (fresh and at 0h, 2h, 4h and 24h post-thaw). Figure S1. Immunophenotyping of fresh cells: Representative histograms of expression of CD90, CD105, CD73 and CD14, CD20, CD34, CD45 and HLA-DR all linked to PerCP. Black peaks represent isotype controls in all histograms. The first row of histograms is for M4 (red), the second for M6 (blue) and the third for M7 (green). All measurements were done in triplicates from three independent experiments based on at least 100,000 events. Figure S2. Immunophenotyping of cryopreserved cells at 0 h post-thaw: Representative histograms of expression of CD90, CD105, CD73 and CD14, CD20, CD34, CD45 and HLA-DR all linked to PerCP. Black peaks represent isotype controls in all histograms. The first row of histograms is for M4 (red), the second for M6 (blue) and the third for M7 (green). All measurements were done in triplicates from three independent experiments based on at least 100,000 events. Figure S3. Immunophenotyping of cryopreserved cells at 2 h post-thaw: Representative histograms of expression of CD90, CD105, CD73 and CD14, CD20, CD34, CD45 and HLA-DR all linked to PerCP. Black peaks represent isotype controls in all histograms. The first row of histograms is for M4 (red), the second for M6 (blue) and the third for M7 (green). All measurements were done in triplicates from three independent experiments based on at least 100,000 events. Figure S4. Immunophenotyping of cryopreserved cells at 4 h post-thaw: Representative histograms of expression of CD90, CD105, CD73 and CD14, CD20, CD34, CD45 and HLA-DR all linked to PerCP. Black peaks represent isotype controls in all histograms. The first row of histograms is for M4 (red), the second for M6 (blue) and the third for M7 (green). All measurements were done in triplicates from three independent experiments based on at least 100,000 events. Figure S [file 13287_2020_2054_MOESM1_ESM.zip › Supplementary 1.pdf]

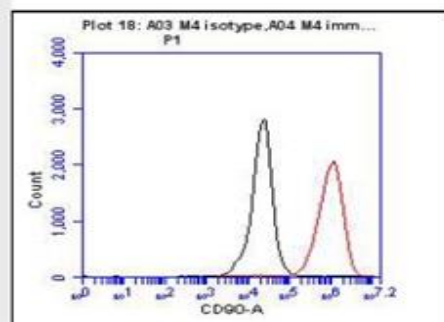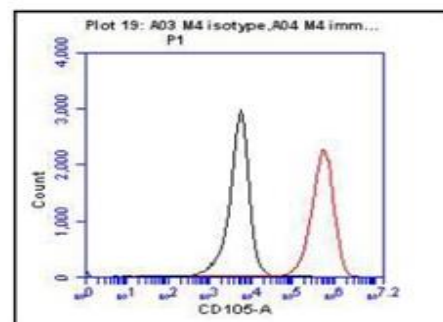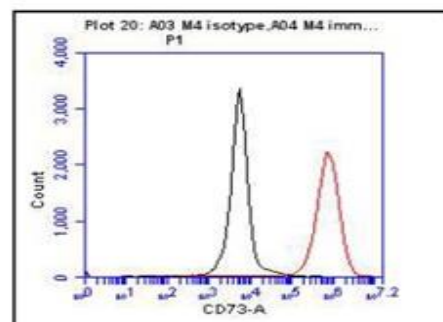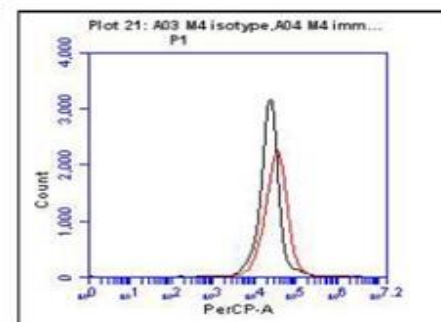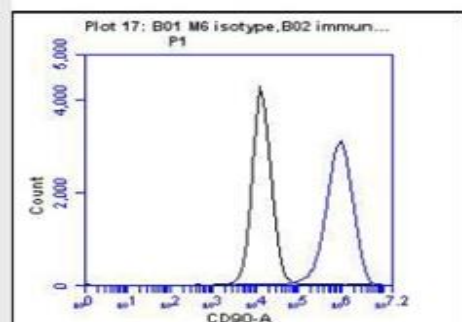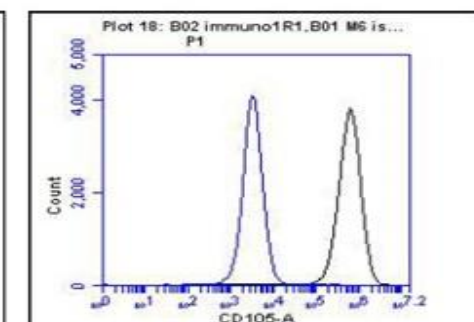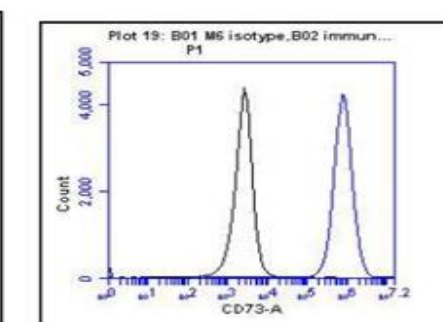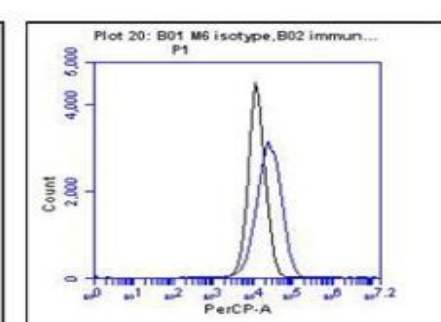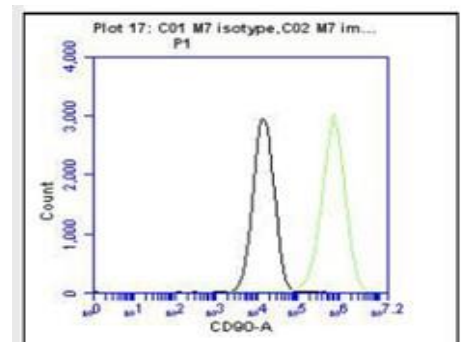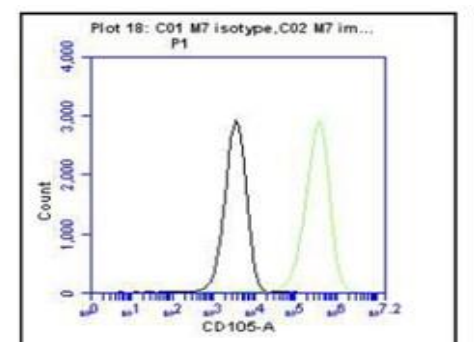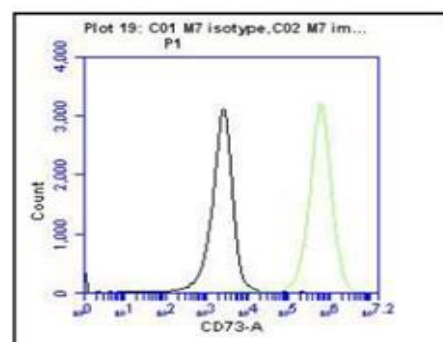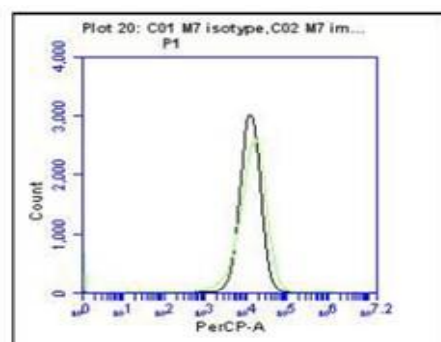

Supplement: Supplementary file 1 — Additional file 1: Figures S1-S8. Representative histograms for immunophenotyping, viability and apoptosis measures for the three lines (fresh and at 0h, 2h, 4h and 24h post-thaw). Figure S1. Immunophenotyping of fresh cells: Representative histograms of expression of CD90, CD105, CD73 and CD14, CD20, CD34, CD45 and HLA-DR all linked to PerCP. Black peaks represent isotype controls in all histograms. The first row of histograms is for M4 (red), the second for M6 (blue) and the third for M7 (green). All measurements were done in triplicates from three independent experiments based on at least 100,000 events. Figure S2. Immunophenotyping of cryopreserved cells at 0 h post-thaw: Representative histograms of expression of CD90, CD105, CD73 and CD14, CD20, CD34, CD45 and HLA-DR all linked to PerCP. Black peaks represent isotype controls in all histograms. The first row of histograms is for M4 (red), the second for M6 (blue) and the third for M7 (green). All measurements were done in triplicates from three independent experiments based on at least 100,000 events. Figure S3. Immunophenotyping of cryopreserved cells at 2 h post-thaw: Representative histograms of expression of CD90, CD105, CD73 and CD14, CD20, CD34, CD45 and HLA-DR all linked to PerCP. Black peaks represent isotype controls in all histograms. The first row of histograms is for M4 (red), the second for M6 (blue) and the third for M7 (green). All measurements were done in triplicates from three independent experiments based on at least 100,000 events. Figure S4. Immunophenotyping of cryopreserved cells at 4 h post-thaw: Representative histograms of expression of CD90, CD105, CD73 and CD14, CD20, CD34, CD45 and HLA-DR all linked to PerCP. Black peaks represent isotype controls in all histograms. The first row of histograms is for M4 (red), the second for M6 (blue) and the third for M7 (green). All measurements were done in triplicates from three independent experiments based on at least 100,000 events. Figure S [file 13287_2020_2054_MOESM1_ESM.zip › Supplementary 2.pdf]

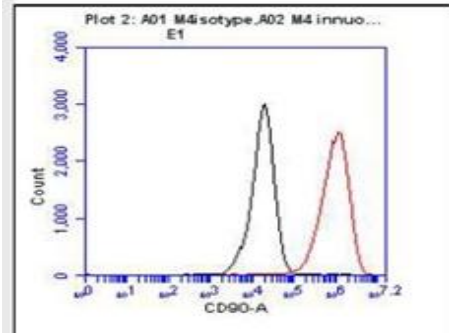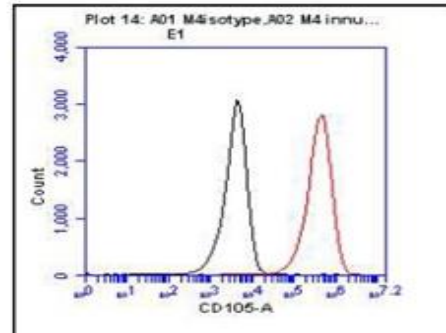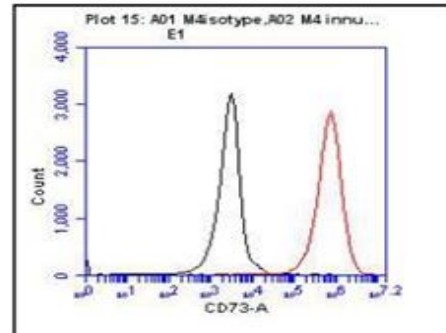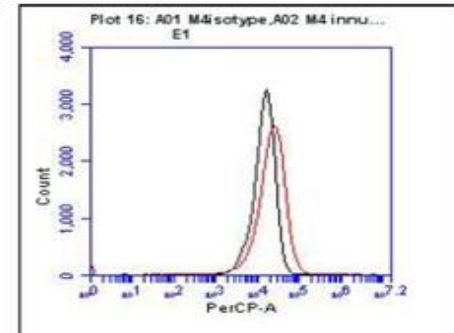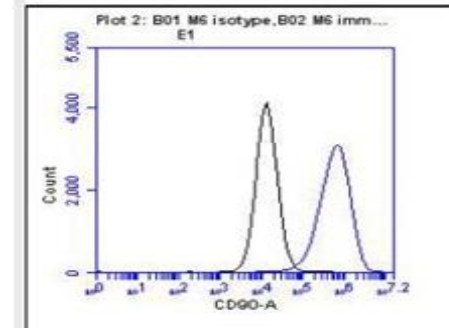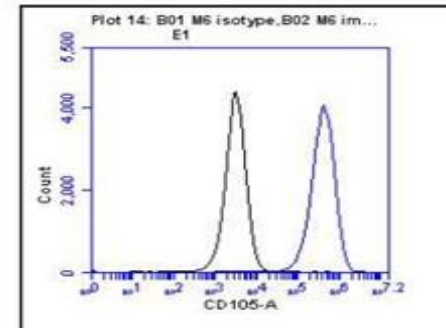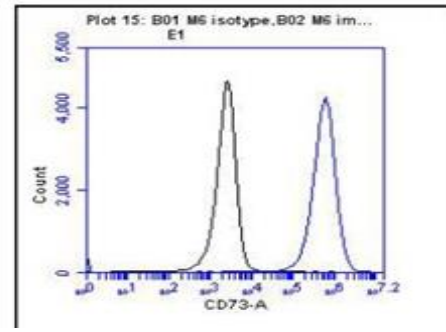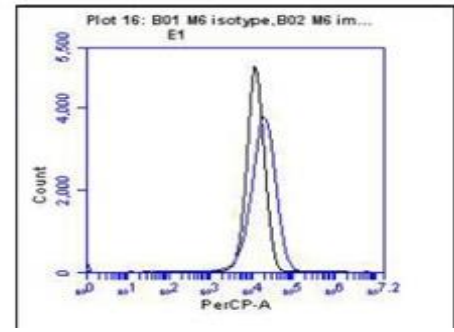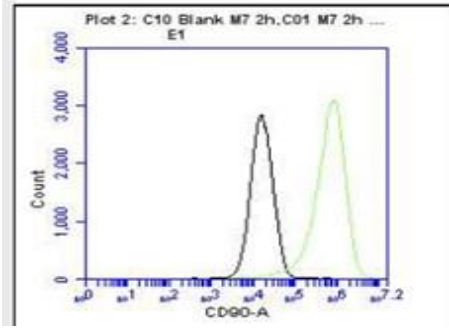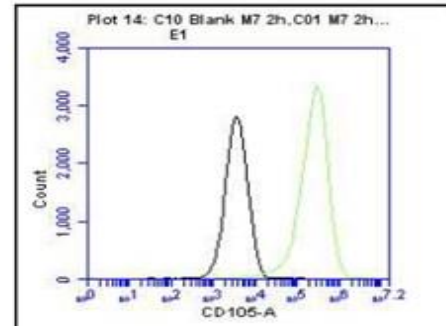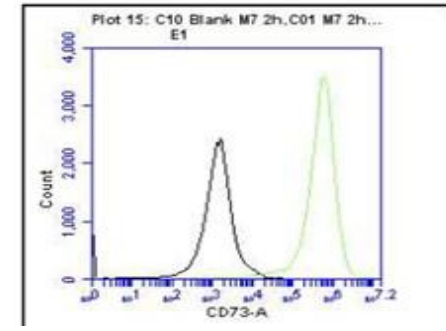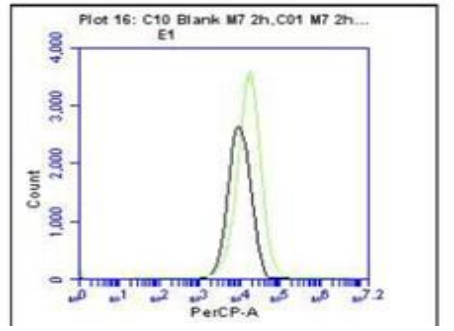

Supplement: Supplementary file 1 — Additional file 1: Figures S1-S8. Representative histograms for immunophenotyping, viability and apoptosis measures for the three lines (fresh and at 0h, 2h, 4h and 24h post-thaw). Figure S1. Immunophenotyping of fresh cells: Representative histograms of expression of CD90, CD105, CD73 and CD14, CD20, CD34, CD45 and HLA-DR all linked to PerCP. Black peaks represent isotype controls in all histograms. The first row of histograms is for M4 (red), the second for M6 (blue) and the third for M7 (green). All measurements were done in triplicates from three independent experiments based on at least 100,000 events. Figure S2. Immunophenotyping of cryopreserved cells at 0 h post-thaw: Representative histograms of expression of CD90, CD105, CD73 and CD14, CD20, CD34, CD45 and HLA-DR all linked to PerCP. Black peaks represent isotype controls in all histograms. The first row of histograms is for M4 (red), the second for M6 (blue) and the third for M7 (green). All measurements were done in triplicates from three independent experiments based on at least 100,000 events. Figure S3. Immunophenotyping of cryopreserved cells at 2 h post-thaw: Representative histograms of expression of CD90, CD105, CD73 and CD14, CD20, CD34, CD45 and HLA-DR all linked to PerCP. Black peaks represent isotype controls in all histograms. The first row of histograms is for M4 (red), the second for M6 (blue) and the third for M7 (green). All measurements were done in triplicates from three independent experiments based on at least 100,000 events. Figure S4. Immunophenotyping of cryopreserved cells at 4 h post-thaw: Representative histograms of expression of CD90, CD105, CD73 and CD14, CD20, CD34, CD45 and HLA-DR all linked to PerCP. Black peaks represent isotype controls in all histograms. The first row of histograms is for M4 (red), the second for M6 (blue) and the third for M7 (green). All measurements were done in triplicates from three independent experiments based on at least 100,000 events. Figure S [file 13287_2020_2054_MOESM1_ESM.zip › Supplementary 3.pdf]

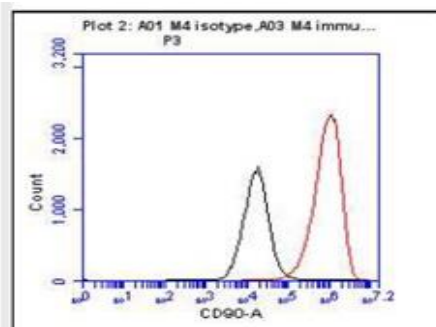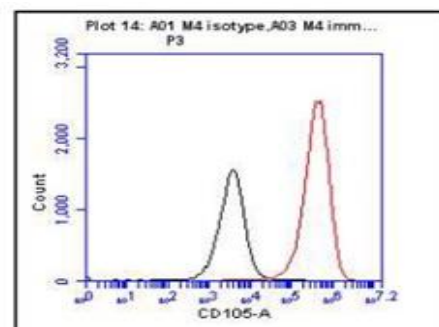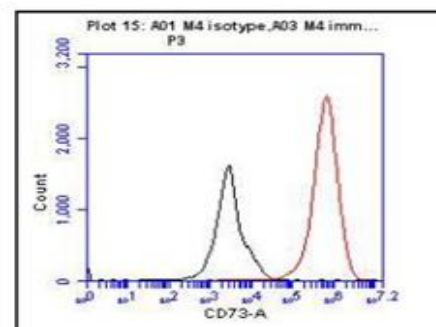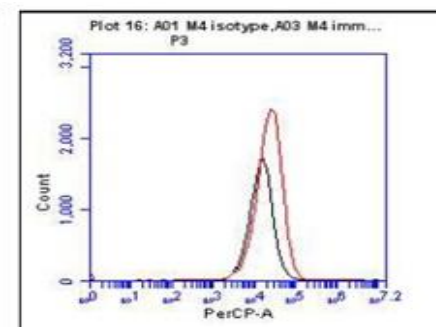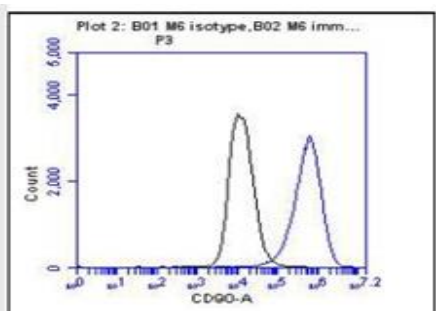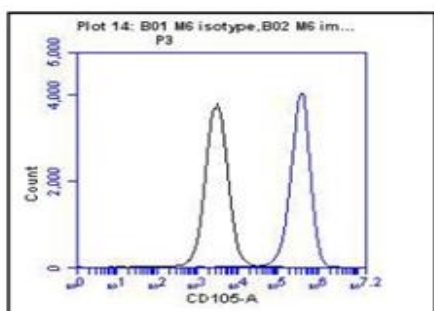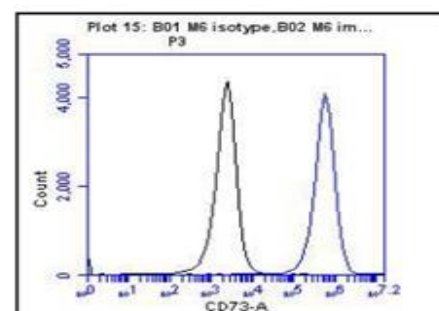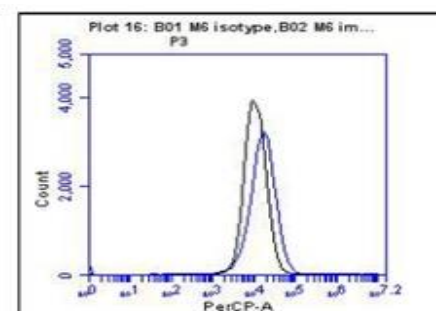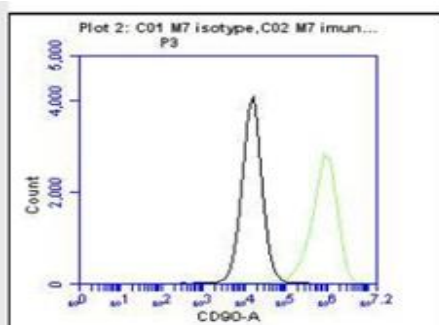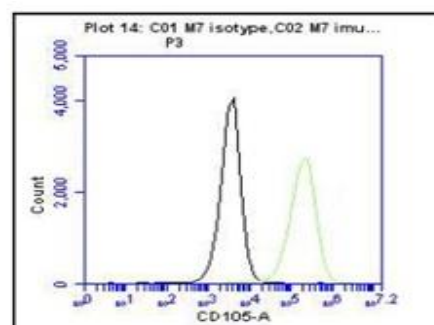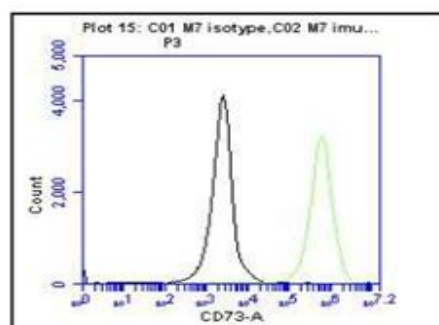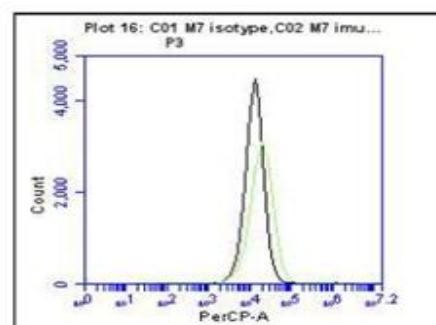

Supplement: Supplementary file 1 — Additional file 1: Figures S1-S8. Representative histograms for immunophenotyping, viability and apoptosis measures for the three lines (fresh and at 0h, 2h, 4h and 24h post-thaw). Figure S1. Immunophenotyping of fresh cells: Representative histograms of expression of CD90, CD105, CD73 and CD14, CD20, CD34, CD45 and HLA-DR all linked to PerCP. Black peaks represent isotype controls in all histograms. The first row of histograms is for M4 (red), the second for M6 (blue) and the third for M7 (green). All measurements were done in triplicates from three independent experiments based on at least 100,000 events. Figure S2. Immunophenotyping of cryopreserved cells at 0 h post-thaw: Representative histograms of expression of CD90, CD105, CD73 and CD14, CD20, CD34, CD45 and HLA-DR all linked to PerCP. Black peaks represent isotype controls in all histograms. The first row of histograms is for M4 (red), the second for M6 (blue) and the third for M7 (green). All measurements were done in triplicates from three independent experiments based on at least 100,000 events. Figure S3. Immunophenotyping of cryopreserved cells at 2 h post-thaw: Representative histograms of expression of CD90, CD105, CD73 and CD14, CD20, CD34, CD45 and HLA-DR all linked to PerCP. Black peaks represent isotype controls in all histograms. The first row of histograms is for M4 (red), the second for M6 (blue) and the third for M7 (green). All measurements were done in triplicates from three independent experiments based on at least 100,000 events. Figure S4. Immunophenotyping of cryopreserved cells at 4 h post-thaw: Representative histograms of expression of CD90, CD105, CD73 and CD14, CD20, CD34, CD45 and HLA-DR all linked to PerCP. Black peaks represent isotype controls in all histograms. The first row of histograms is for M4 (red), the second for M6 (blue) and the third for M7 (green). All measurements were done in triplicates from three independent experiments based on at least 100,000 events. Figure S [file 13287_2020_2054_MOESM1_ESM.zip › Supplementary 4.pdf]

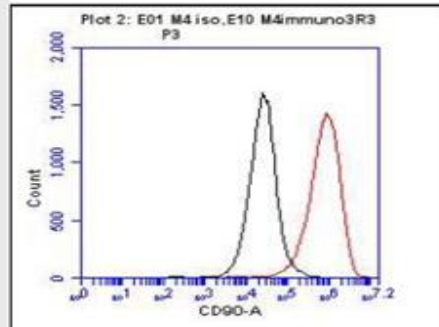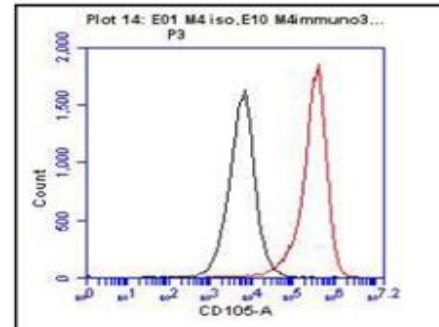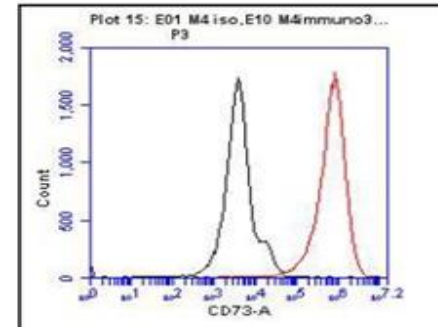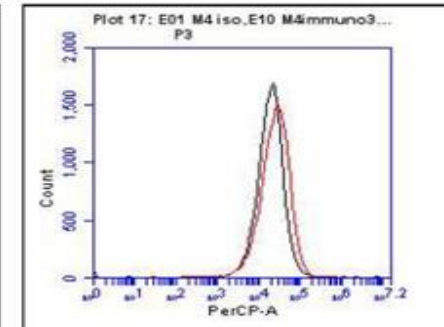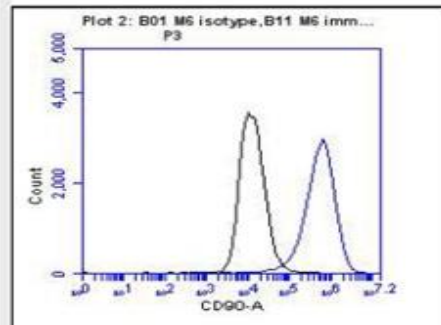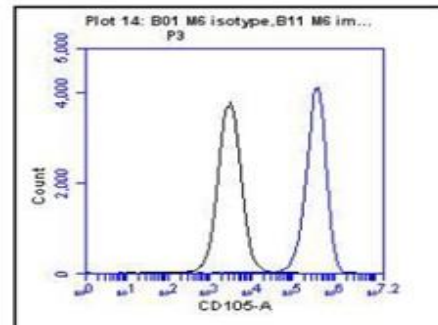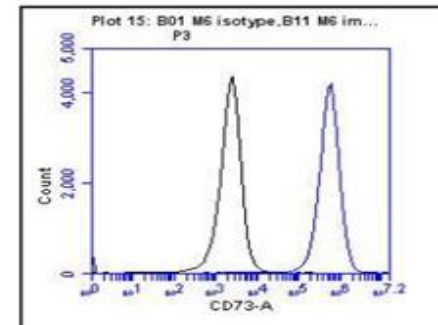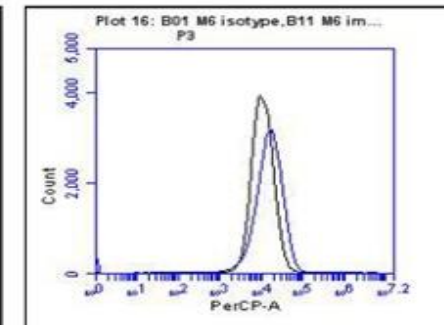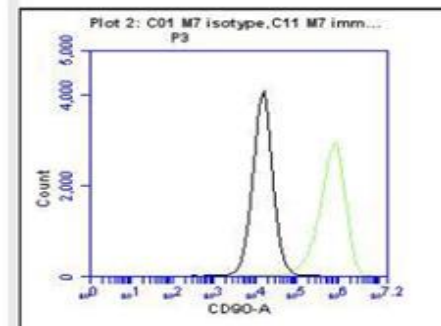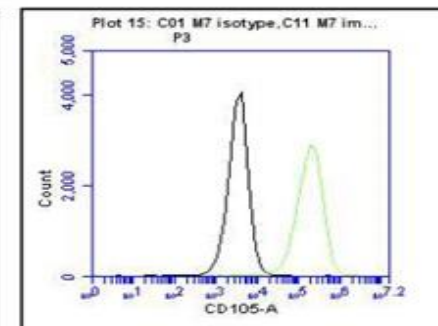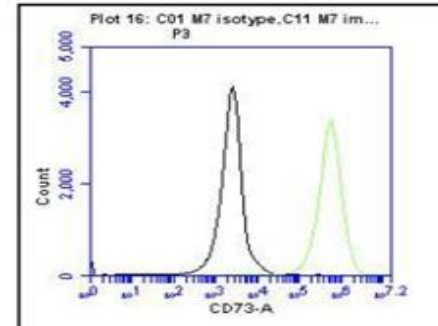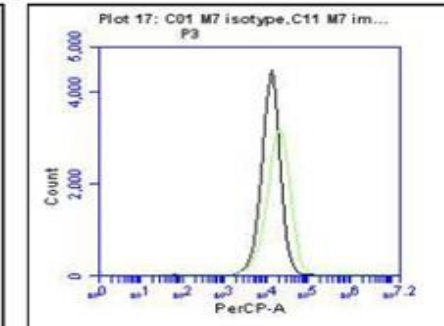

Supplement: Supplementary file 1 — Additional file 1: Figures S1-S8. Representative histograms for immunophenotyping, viability and apoptosis measures for the three lines (fresh and at 0h, 2h, 4h and 24h post-thaw). Figure S1. Immunophenotyping of fresh cells: Representative histograms of expression of CD90, CD105, CD73 and CD14, CD20, CD34, CD45 and HLA-DR all linked to PerCP. Black peaks represent isotype controls in all histograms. The first row of histograms is for M4 (red), the second for M6 (blue) and the third for M7 (green). All measurements were done in triplicates from three independent experiments based on at least 100,000 events. Figure S2. Immunophenotyping of cryopreserved cells at 0 h post-thaw: Representative histograms of expression of CD90, CD105, CD73 and CD14, CD20, CD34, CD45 and HLA-DR all linked to PerCP. Black peaks represent isotype controls in all histograms. The first row of histograms is for M4 (red), the second for M6 (blue) and the third for M7 (green). All measurements were done in triplicates from three independent experiments based on at least 100,000 events. Figure S3. Immunophenotyping of cryopreserved cells at 2 h post-thaw: Representative histograms of expression of CD90, CD105, CD73 and CD14, CD20, CD34, CD45 and HLA-DR all linked to PerCP. Black peaks represent isotype controls in all histograms. The first row of histograms is for M4 (red), the second for M6 (blue) and the third for M7 (green). All measurements were done in triplicates from three independent experiments based on at least 100,000 events. Figure S4. Immunophenotyping of cryopreserved cells at 4 h post-thaw: Representative histograms of expression of CD90, CD105, CD73 and CD14, CD20, CD34, CD45 and HLA-DR all linked to PerCP. Black peaks represent isotype controls in all histograms. The first row of histograms is for M4 (red), the second for M6 (blue) and the third for M7 (green). All measurements were done in triplicates from three independent experiments based on at least 100,000 events. Figure S [file 13287_2020_2054_MOESM1_ESM.zip › Supplementary 5.pdf]

**Fresh**

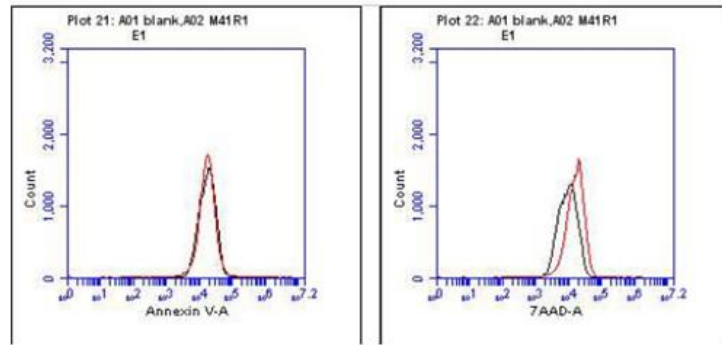

**0h**

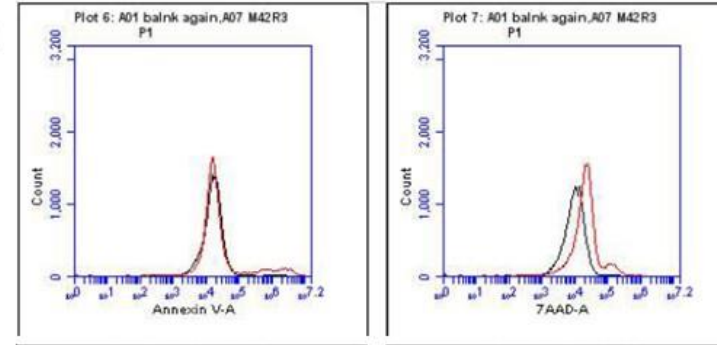

**2h**

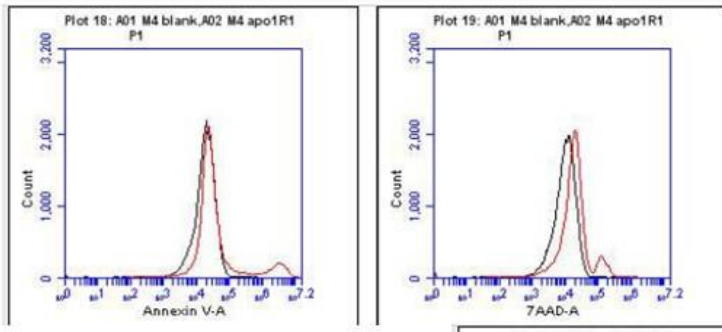

**4h**

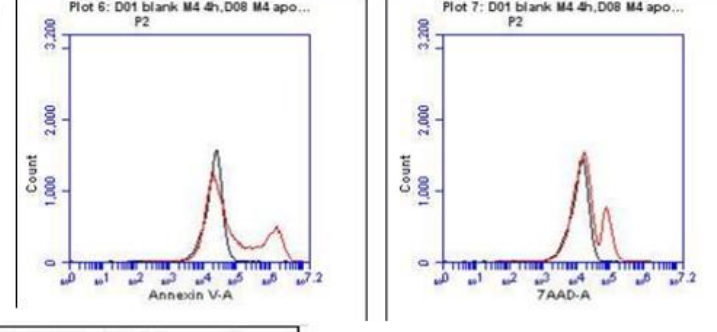

**24h**

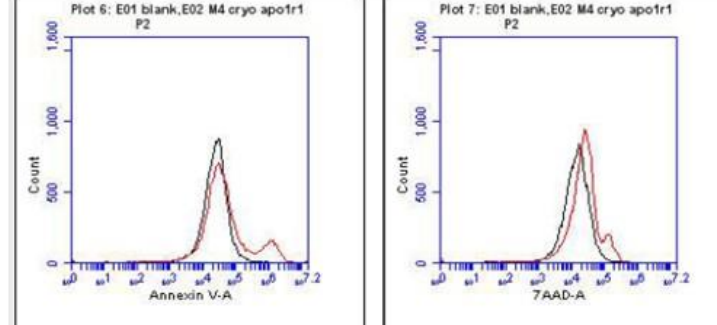

Supplement: Supplementary file 1 — Additional file 1: Figures S1-S8. Representative histograms for immunophenotyping, viability and apoptosis measures for the three lines (fresh and at 0h, 2h, 4h and 24h post-thaw). Figure S1. Immunophenotyping of fresh cells: Representative histograms of expression of CD90, CD105, CD73 and CD14, CD20, CD34, CD45 and HLA-DR all linked to PerCP. Black peaks represent isotype controls in all histograms. The first row of histograms is for M4 (red), the second for M6 (blue) and the third for M7 (green). All measurements were done in triplicates from three independent experiments based on at least 100,000 events. Figure S2. Immunophenotyping of cryopreserved cells at 0 h post-thaw: Representative histograms of expression of CD90, CD105, CD73 and CD14, CD20, CD34, CD45 and HLA-DR all linked to PerCP. Black peaks represent isotype controls in all histograms. The first row of histograms is for M4 (red), the second for M6 (blue) and the third for M7 (green). All measurements were done in triplicates from three independent experiments based on at least 100,000 events. Figure S3. Immunophenotyping of cryopreserved cells at 2 h post-thaw: Representative histograms of expression of CD90, CD105, CD73 and CD14, CD20, CD34, CD45 and HLA-DR all linked to PerCP. Black peaks represent isotype controls in all histograms. The first row of histograms is for M4 (red), the second for M6 (blue) and the third for M7 (green). All measurements were done in triplicates from three independent experiments based on at least 100,000 events. Figure S4. Immunophenotyping of cryopreserved cells at 4 h post-thaw: Representative histograms of expression of CD90, CD105, CD73 and CD14, CD20, CD34, CD45 and HLA-DR all linked to PerCP. Black peaks represent isotype controls in all histograms. The first row of histograms is for M4 (red), the second for M6 (blue) and the third for M7 (green). All measurements were done in triplicates from three independent experiments based on at least 100,000 events. Figure S [file 13287_2020_2054_MOESM1_ESM.zip › Supplementary 6.pdf]

**Fresh**

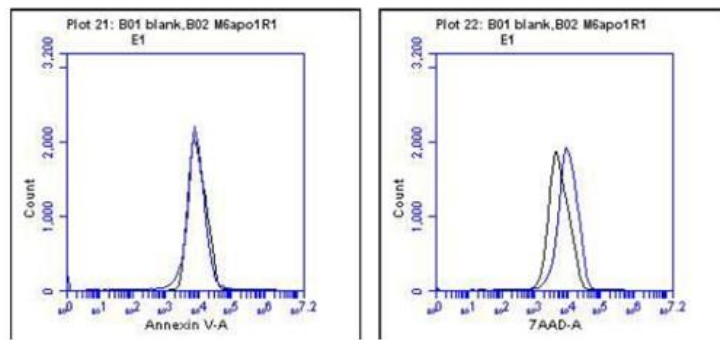

**0h**

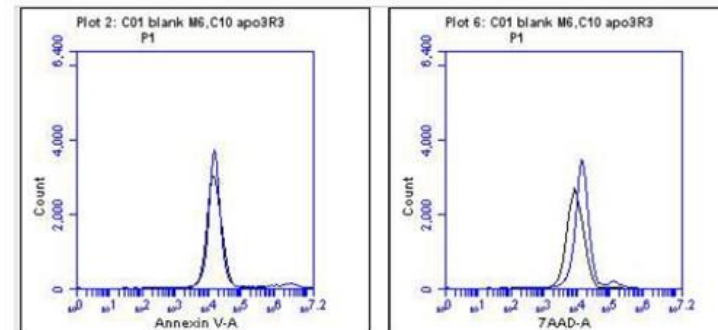

**2h**

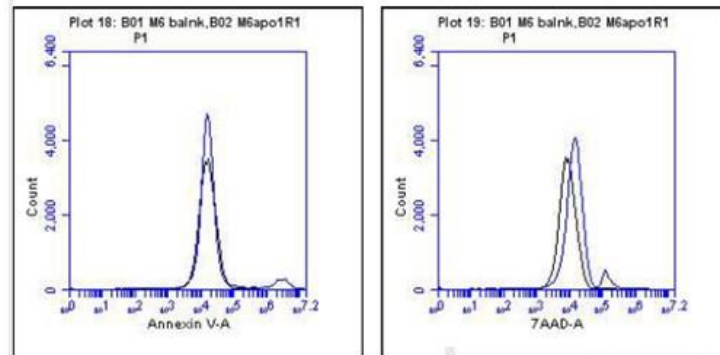

**4h**

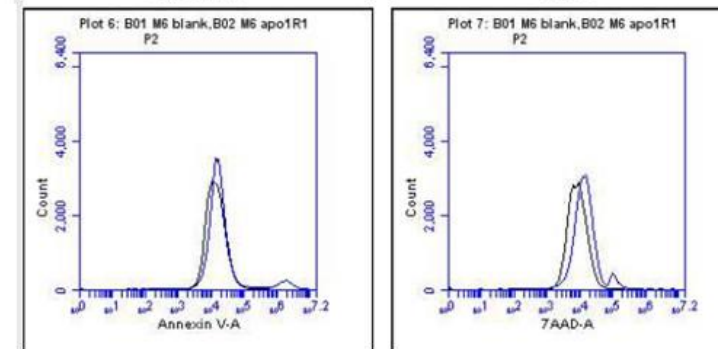

**24h**

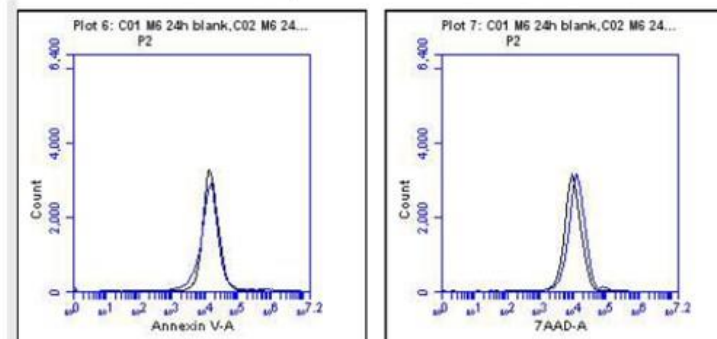

Supplement: Supplementary file 1 — Additional file 1: Figures S1-S8. Representative histograms for immunophenotyping, viability and apoptosis measures for the three lines (fresh and at 0h, 2h, 4h and 24h post-thaw). Figure S1. Immunophenotyping of fresh cells: Representative histograms of expression of CD90, CD105, CD73 and CD14, CD20, CD34, CD45 and HLA-DR all linked to PerCP. Black peaks represent isotype controls in all histograms. The first row of histograms is for M4 (red), the second for M6 (blue) and the third for M7 (green). All measurements were done in triplicates from three independent experiments based on at least 100,000 events. Figure S2. Immunophenotyping of cryopreserved cells at 0 h post-thaw: Representative histograms of expression of CD90, CD105, CD73 and CD14, CD20, CD34, CD45 and HLA-DR all linked to PerCP. Black peaks represent isotype controls in all histograms. The first row of histograms is for M4 (red), the second for M6 (blue) and the third for M7 (green). All measurements were done in triplicates from three independent experiments based on at least 100,000 events. Figure S3. Immunophenotyping of cryopreserved cells at 2 h post-thaw: Representative histograms of expression of CD90, CD105, CD73 and CD14, CD20, CD34, CD45 and HLA-DR all linked to PerCP. Black peaks represent isotype controls in all histograms. The first row of histograms is for M4 (red), the second for M6 (blue) and the third for M7 (green). All measurements were done in triplicates from three independent experiments based on at least 100,000 events. Figure S4. Immunophenotyping of cryopreserved cells at 4 h post-thaw: Representative histograms of expression of CD90, CD105, CD73 and CD14, CD20, CD34, CD45 and HLA-DR all linked to PerCP. Black peaks represent isotype controls in all histograms. The first row of histograms is for M4 (red), the second for M6 (blue) and the third for M7 (green). All measurements were done in triplicates from three independent experiments based on at least 100,000 events. Figure S [file 13287_2020_2054_MOESM1_ESM.zip › Supplementary 7.pdf]

**Fresh**

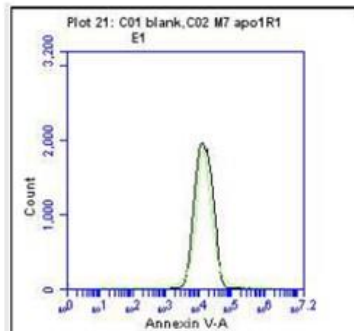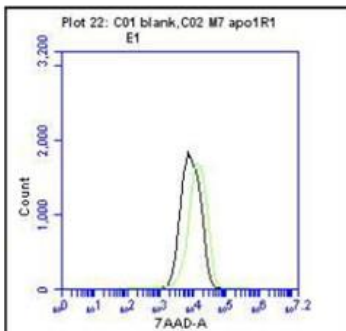

**2h**

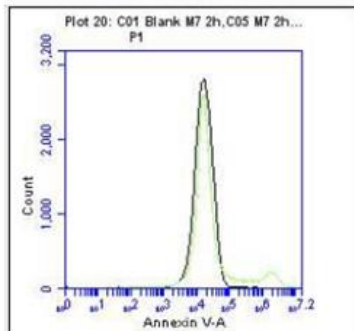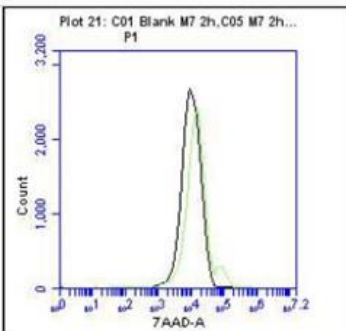

**0h**

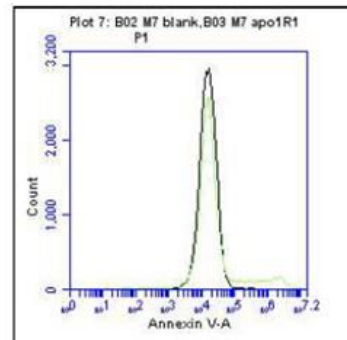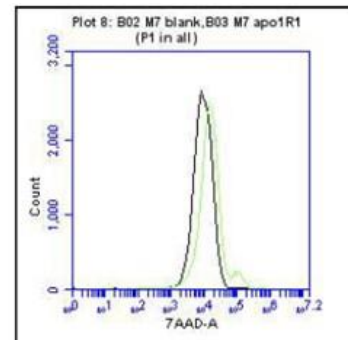

**4h**

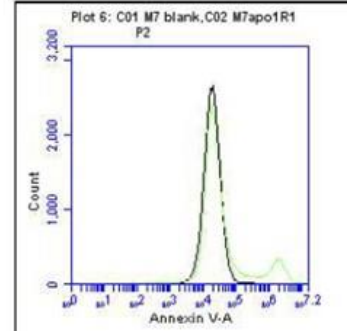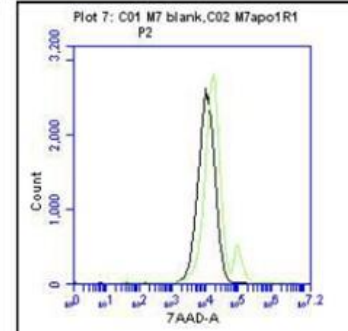

**24h**

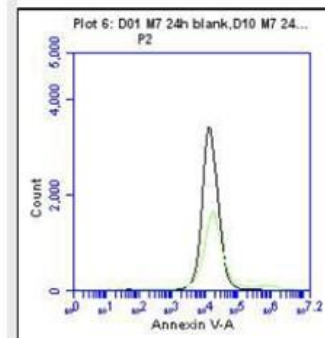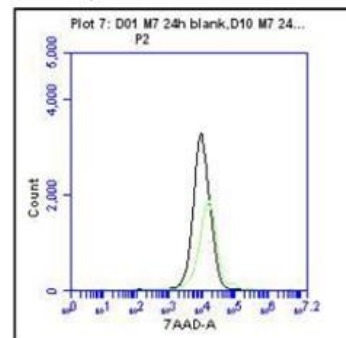

Supplement: Supplementary file 1 — Additional file 1: Figures S1-S8. Representative histograms for immunophenotyping, viability and apoptosis measures for the three lines (fresh and at 0h, 2h, 4h and 24h post-thaw). Figure S1. Immunophenotyping of fresh cells: Representative histograms of expression of CD90, CD105, CD73 and CD14, CD20, CD34, CD45 and HLA-DR all linked to PerCP. Black peaks represent isotype controls in all histograms. The first row of histograms is for M4 (red), the second for M6 (blue) and the third for M7 (green). All measurements were done in triplicates from three independent experiments based on at least 100,000 events. Figure S2. Immunophenotyping of cryopreserved cells at 0 h post-thaw: Representative histograms of expression of CD90, CD105, CD73 and CD14, CD20, CD34, CD45 and HLA-DR all linked to PerCP. Black peaks represent isotype controls in all histograms. The first row of histograms is for M4 (red), the second for M6 (blue) and the third for M7 (green). All measurements were done in triplicates from three independent experiments based on at least 100,000 events. Figure S3. Immunophenotyping of cryopreserved cells at 2 h post-thaw: Representative histograms of expression of CD90, CD105, CD73 and CD14, CD20, CD34, CD45 and HLA-DR all linked to PerCP. Black peaks represent isotype controls in all histograms. The first row of histograms is for M4 (red), the second for M6 (blue) and the third for M7 (green). All measurements were done in triplicates from three independent experiments based on at least 100,000 events. Figure S4. Immunophenotyping of cryopreserved cells at 4 h post-thaw: Representative histograms of expression of CD90, CD105, CD73 and CD14, CD20, CD34, CD45 and HLA-DR all linked to PerCP. Black peaks represent isotype controls in all histograms. The first row of histograms is for M4 (red), the second for M6 (blue) and the third for M7 (green). All measurements were done in triplicates from three independent experiments based on at least 100,000 events. Figure S [file 13287_2020_2054_MOESM1_ESM.zip › Supplementary 8.pdf]

**A**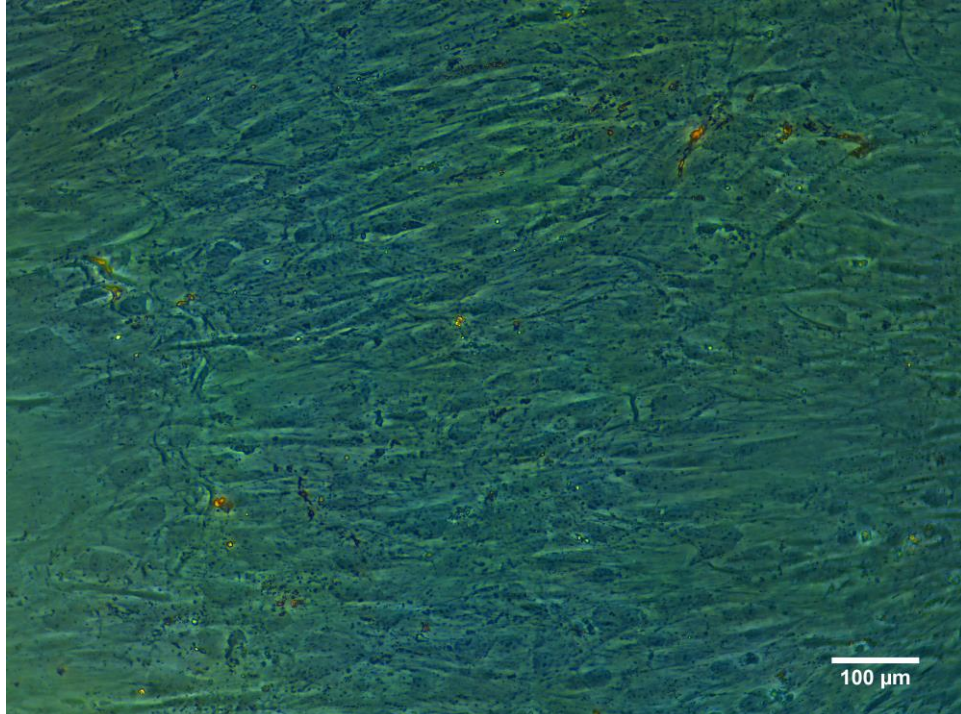**B**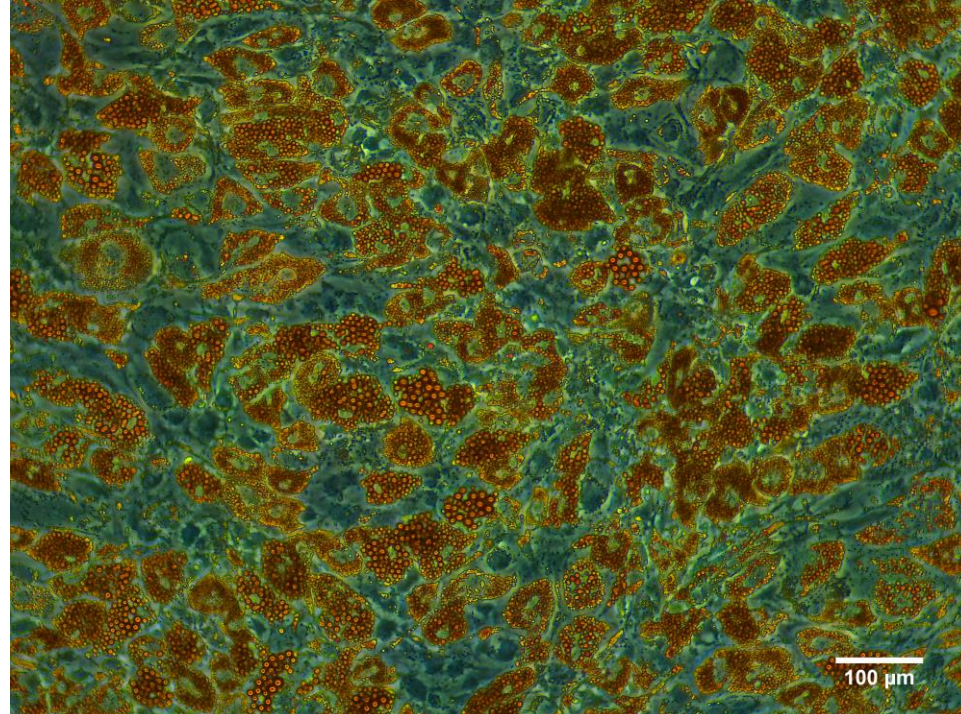**C**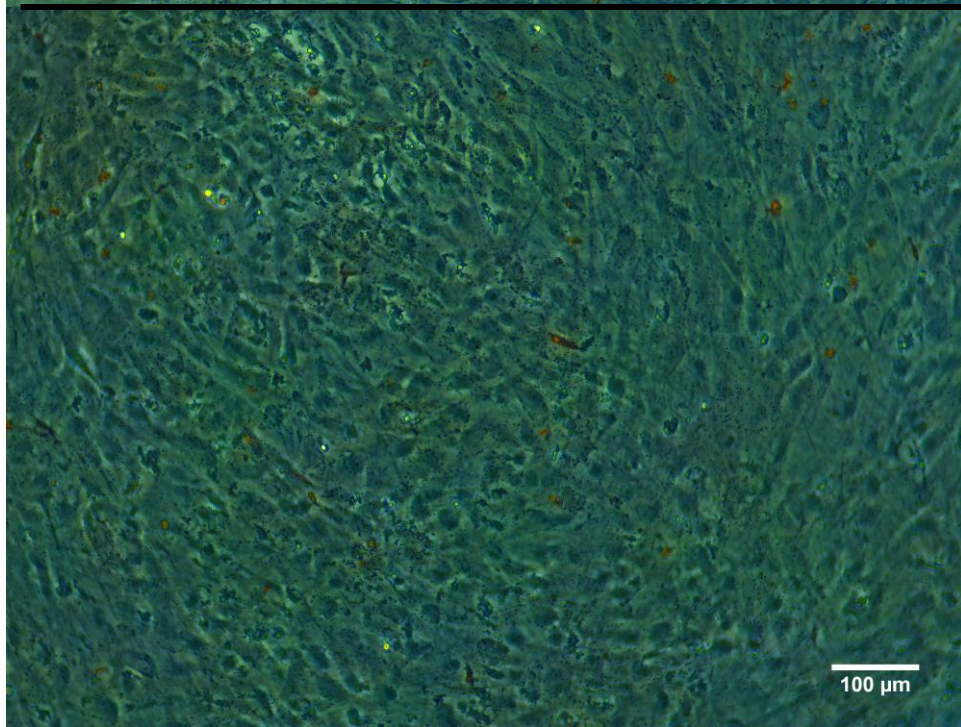**D**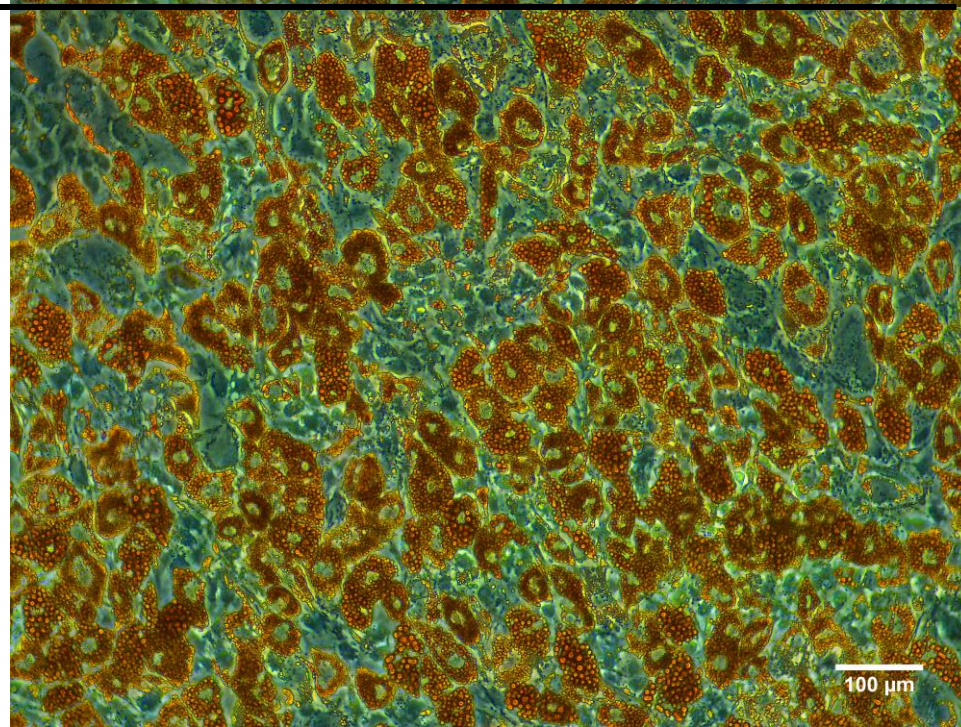

Supplement: Supplementary file 2 — Additional file 2: Figures S9-S15. fresh and cryopreserved cells stained positive for Oil Red O staining and Alizarin Red staining after incubation with adipogenic and osteogenic differentiation media. Figure S9. Oil Red O staining of fresh and cryopreserved M4 cells following 19 days of incubation in adipogenic differentiation media: Light microscopy representative images: (A) M4 fresh control well, (B) M4 fresh differentiation well, (C) M4 cryopreserved control well, (D) M4 cryopreserved differentiation well. Scale bars represent 100μm. Figure S10. Oil Red O staining of fresh and cryopreserved M6 cells following 19 days of incubation in adipogenic differentiation media: Light microscopy representative images: (A) M6 fresh control well, (B) M6 fresh differentiation well, (C) M6 cryopreserved control well, (D) M6 cryopreserved differentiation well. Scale bars represent 100μm. Figure S11. Oil Red O staining of fresh and cryopreserved M7 cells following 19 days of incubation in adipoegnic differentiation media: Light microscopy representative images: (A) M7 fresh control well, (B) M7 fresh differentiation well, (C) M7 cryopreserved control well, (D) M7 cryopreserved differentiation well. Scale bars represent 100μm. Figure S12. Alizarin Red staining of fresh and cryopreserved M4 cells following 16 days of incubation in osteogenic differentiation media: Light microscopy representative images: (A) M4 fresh control well, (B) M4 fresh differentiation well, (C) M4 cryopreserved control well, (D) M4 cryopreserved differentiation well. Scale bars represent 100μm. Figure S13. Alizarin Red staining of fresh and cryopreserved M6 cells following 16 days of incubation with osteogenic differentiation media: Light microscopy representative images: (A) M6 fresh control well, (B) M6 fresh differentiation well, (C) M6 cryopreserved control well, (D) M6 cryopreserved differentiation well. Scale bars represent 100μm. Figure S14. Alizarin Red staining of fresh and cryopreserved M7 cells f [file 13287_2020_2054_MOESM2_ESM.zip › Supplementary 10.pdf]

**A**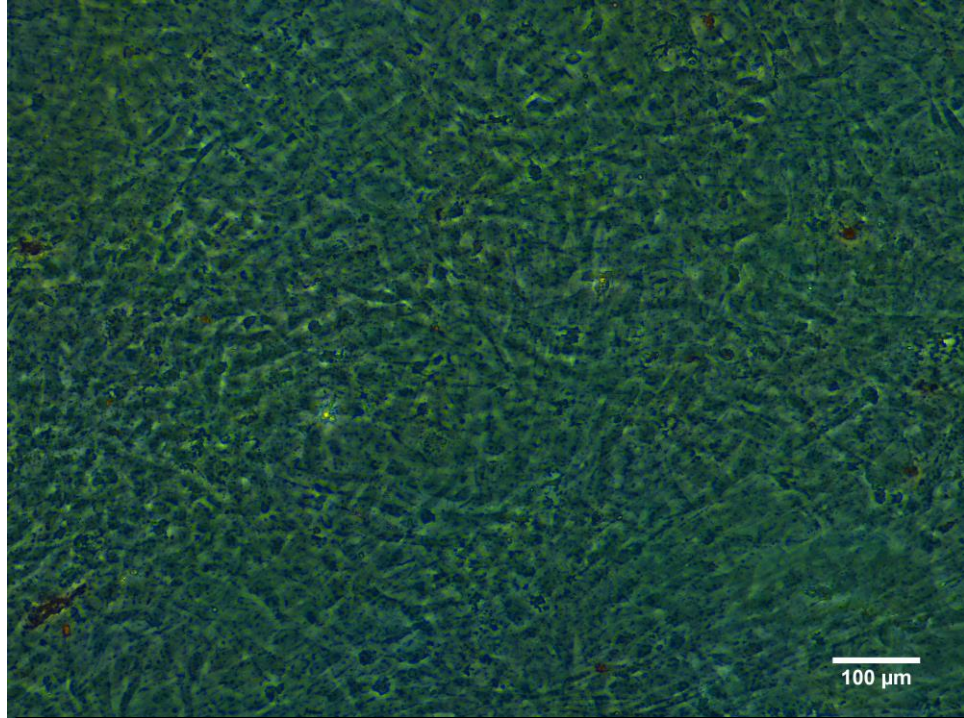**B**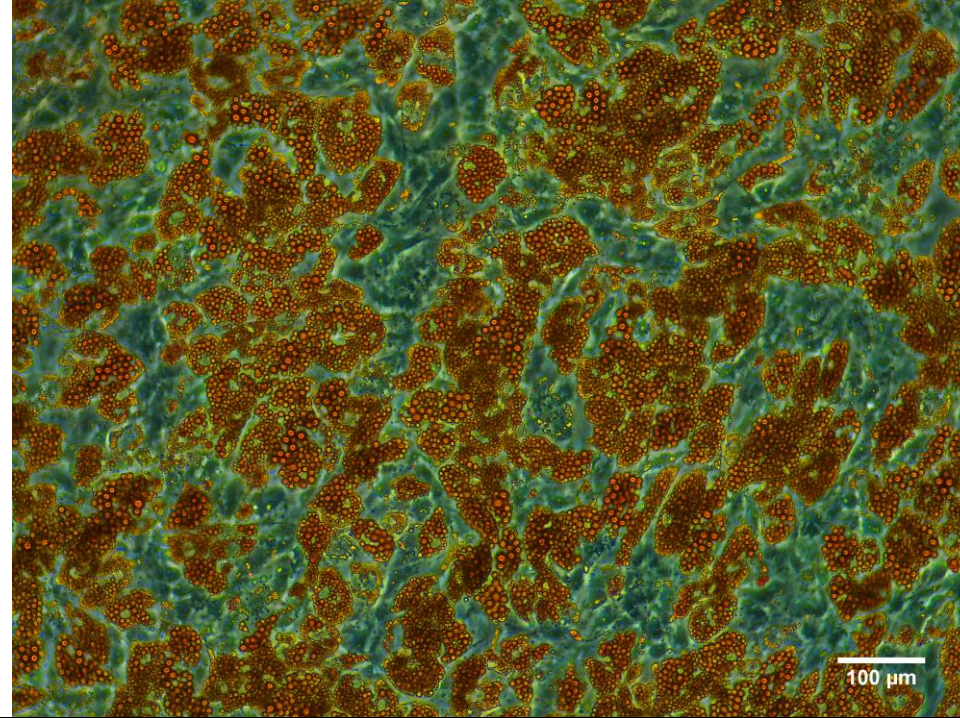**C**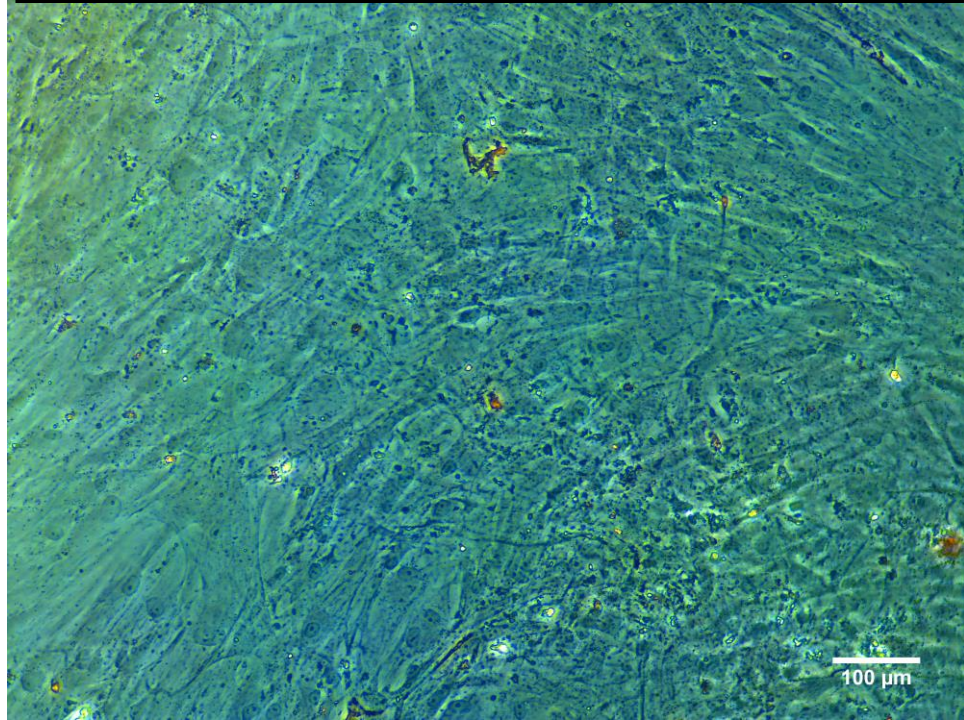**D**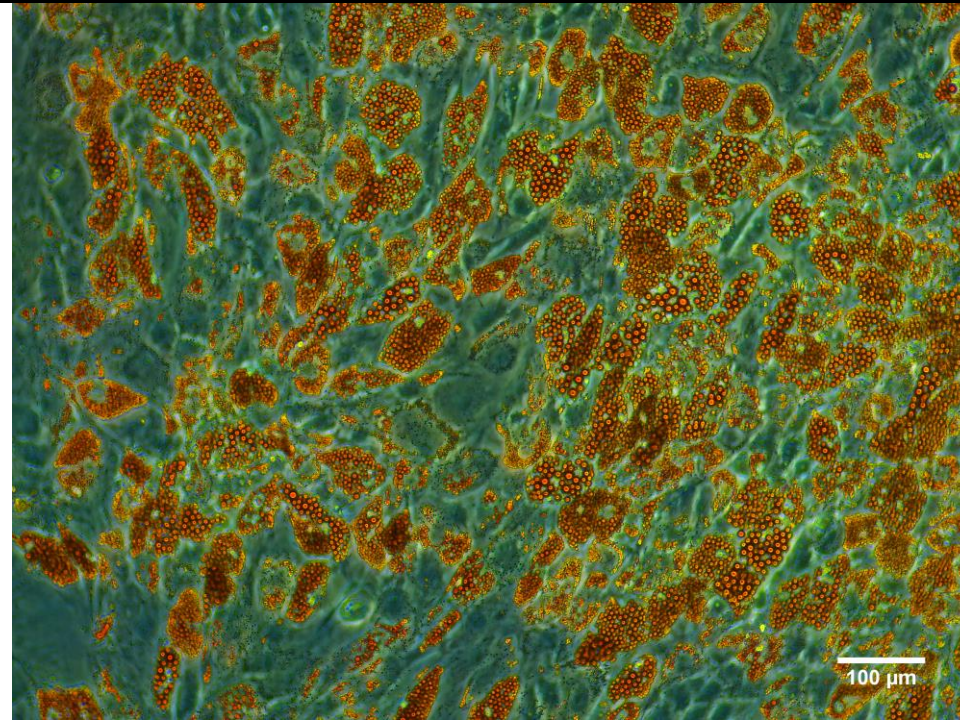

Supplement: Supplementary file 2 — Additional file 2: Figures S9-S15. fresh and cryopreserved cells stained positive for Oil Red O staining and Alizarin Red staining after incubation with adipogenic and osteogenic differentiation media. Figure S9. Oil Red O staining of fresh and cryopreserved M4 cells following 19 days of incubation in adipogenic differentiation media: Light microscopy representative images: (A) M4 fresh control well, (B) M4 fresh differentiation well, (C) M4 cryopreserved control well, (D) M4 cryopreserved differentiation well. Scale bars represent 100μm. Figure S10. Oil Red O staining of fresh and cryopreserved M6 cells following 19 days of incubation in adipogenic differentiation media: Light microscopy representative images: (A) M6 fresh control well, (B) M6 fresh differentiation well, (C) M6 cryopreserved control well, (D) M6 cryopreserved differentiation well. Scale bars represent 100μm. Figure S11. Oil Red O staining of fresh and cryopreserved M7 cells following 19 days of incubation in adipoegnic differentiation media: Light microscopy representative images: (A) M7 fresh control well, (B) M7 fresh differentiation well, (C) M7 cryopreserved control well, (D) M7 cryopreserved differentiation well. Scale bars represent 100μm. Figure S12. Alizarin Red staining of fresh and cryopreserved M4 cells following 16 days of incubation in osteogenic differentiation media: Light microscopy representative images: (A) M4 fresh control well, (B) M4 fresh differentiation well, (C) M4 cryopreserved control well, (D) M4 cryopreserved differentiation well. Scale bars represent 100μm. Figure S13. Alizarin Red staining of fresh and cryopreserved M6 cells following 16 days of incubation with osteogenic differentiation media: Light microscopy representative images: (A) M6 fresh control well, (B) M6 fresh differentiation well, (C) M6 cryopreserved control well, (D) M6 cryopreserved differentiation well. Scale bars represent 100μm. Figure S14. Alizarin Red staining of fresh and cryopreserved M7 cells f [file 13287_2020_2054_MOESM2_ESM.zip › Supplementary 11.pdf]

**A**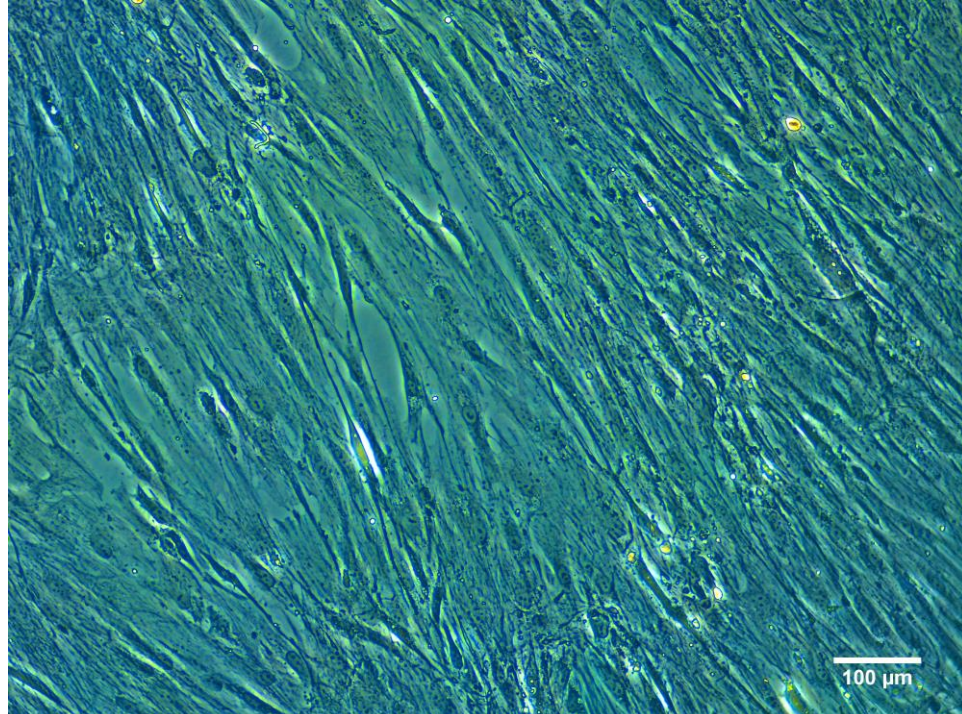**B**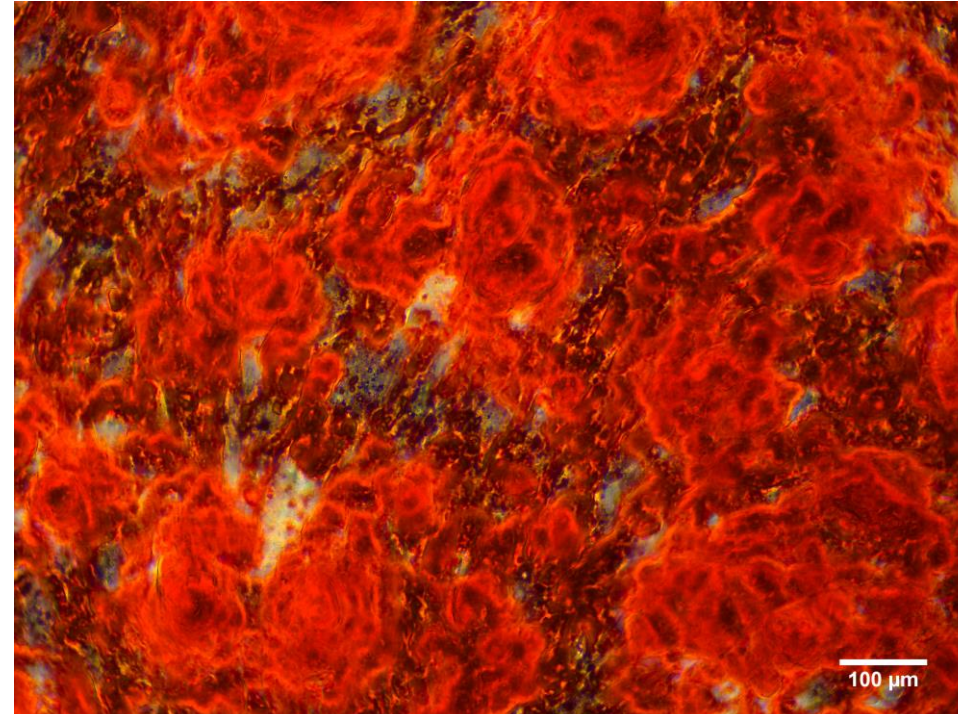**C**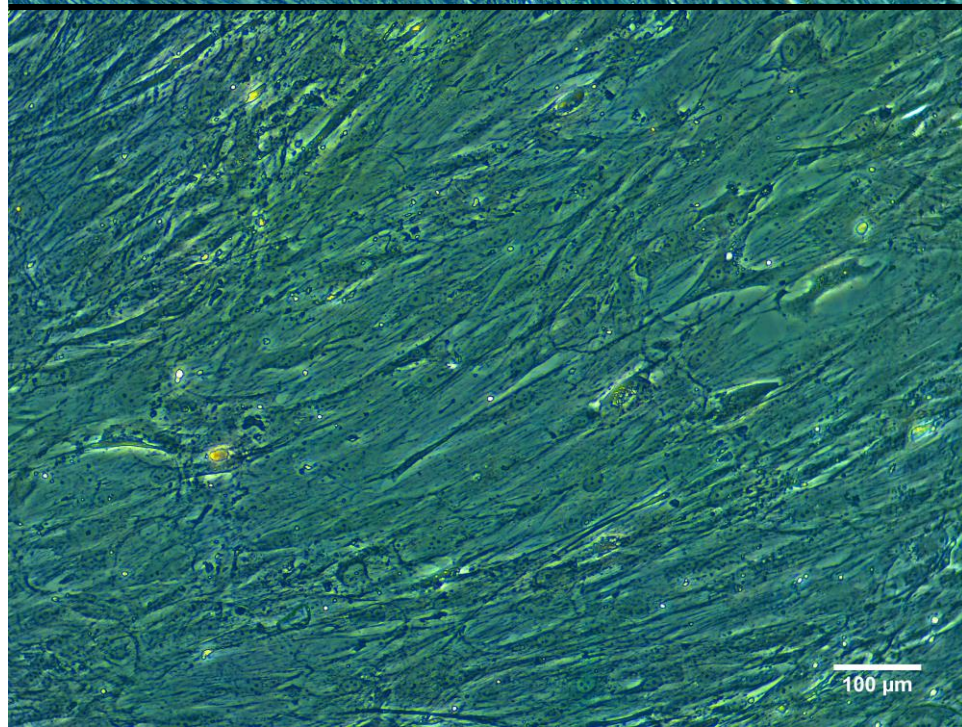**D**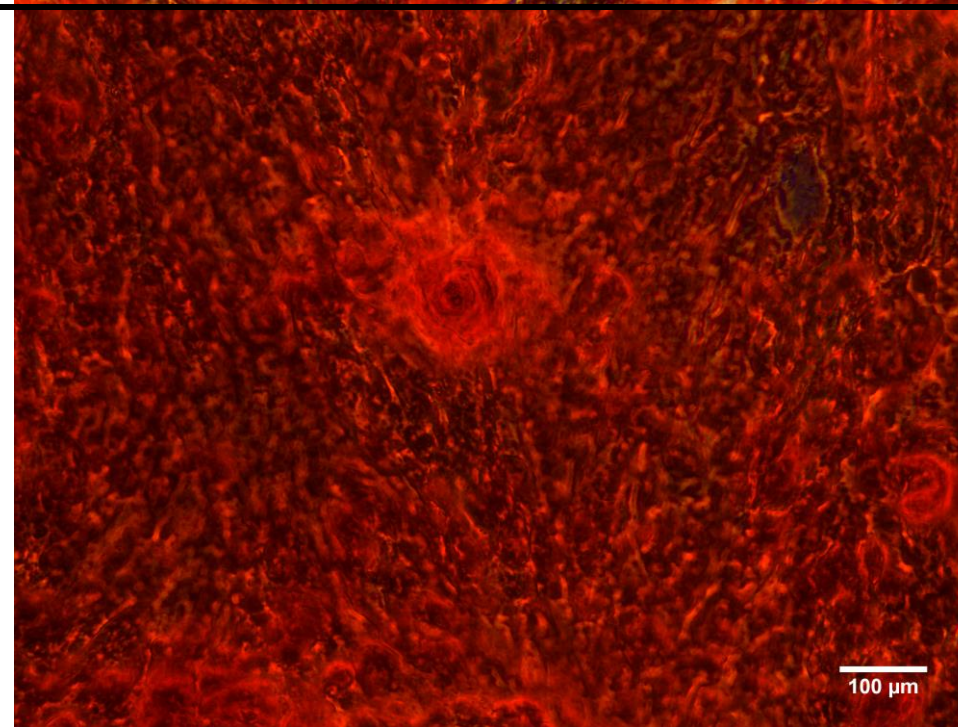

Supplement: Supplementary file 2 — Additional file 2: Figures S9-S15. fresh and cryopreserved cells stained positive for Oil Red O staining and Alizarin Red staining after incubation with adipogenic and osteogenic differentiation media. Figure S9. Oil Red O staining of fresh and cryopreserved M4 cells following 19 days of incubation in adipogenic differentiation media: Light microscopy representative images: (A) M4 fresh control well, (B) M4 fresh differentiation well, (C) M4 cryopreserved control well, (D) M4 cryopreserved differentiation well. Scale bars represent 100μm. Figure S10. Oil Red O staining of fresh and cryopreserved M6 cells following 19 days of incubation in adipogenic differentiation media: Light microscopy representative images: (A) M6 fresh control well, (B) M6 fresh differentiation well, (C) M6 cryopreserved control well, (D) M6 cryopreserved differentiation well. Scale bars represent 100μm. Figure S11. Oil Red O staining of fresh and cryopreserved M7 cells following 19 days of incubation in adipoegnic differentiation media: Light microscopy representative images: (A) M7 fresh control well, (B) M7 fresh differentiation well, (C) M7 cryopreserved control well, (D) M7 cryopreserved differentiation well. Scale bars represent 100μm. Figure S12. Alizarin Red staining of fresh and cryopreserved M4 cells following 16 days of incubation in osteogenic differentiation media: Light microscopy representative images: (A) M4 fresh control well, (B) M4 fresh differentiation well, (C) M4 cryopreserved control well, (D) M4 cryopreserved differentiation well. Scale bars represent 100μm. Figure S13. Alizarin Red staining of fresh and cryopreserved M6 cells following 16 days of incubation with osteogenic differentiation media: Light microscopy representative images: (A) M6 fresh control well, (B) M6 fresh differentiation well, (C) M6 cryopreserved control well, (D) M6 cryopreserved differentiation well. Scale bars represent 100μm. Figure S14. Alizarin Red staining of fresh and cryopreserved M7 cells f [file 13287_2020_2054_MOESM2_ESM.zip › Supplementary 12.pdf]

**A**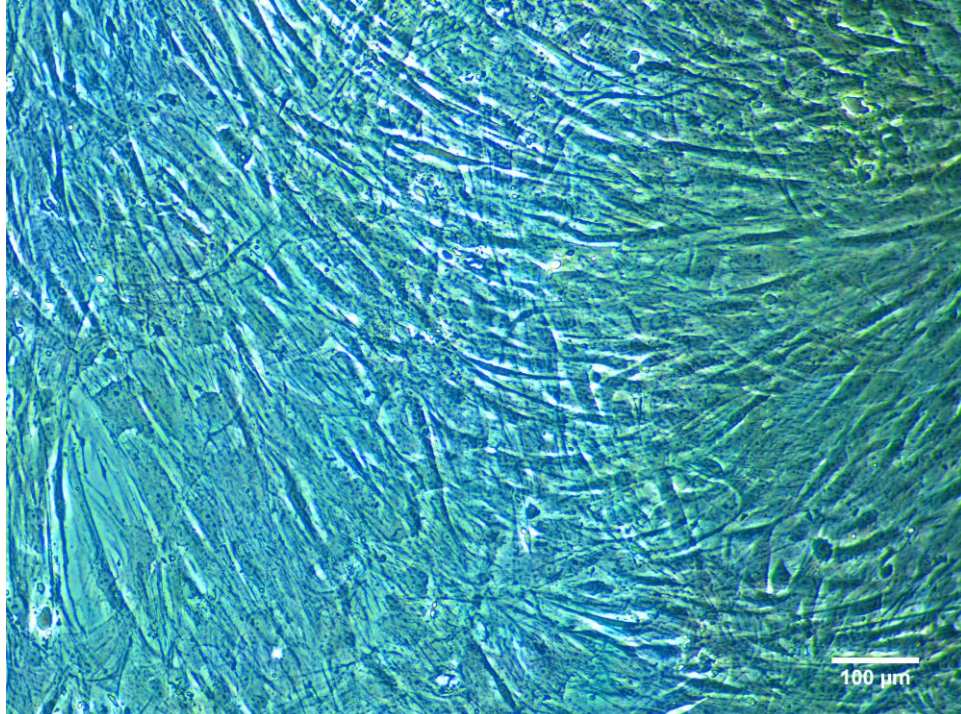**B**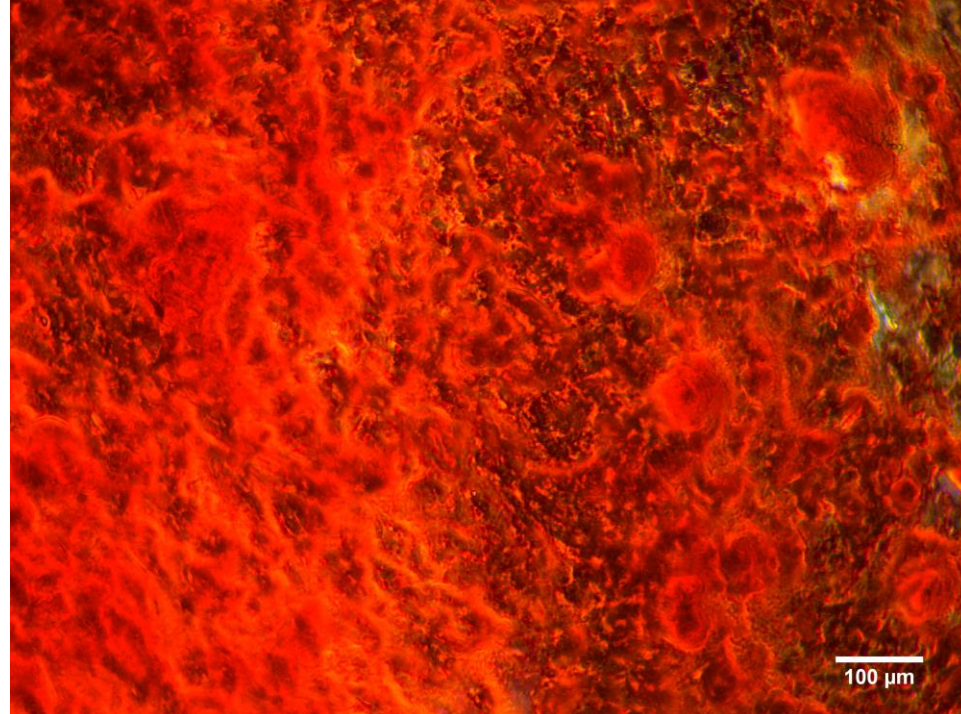**C**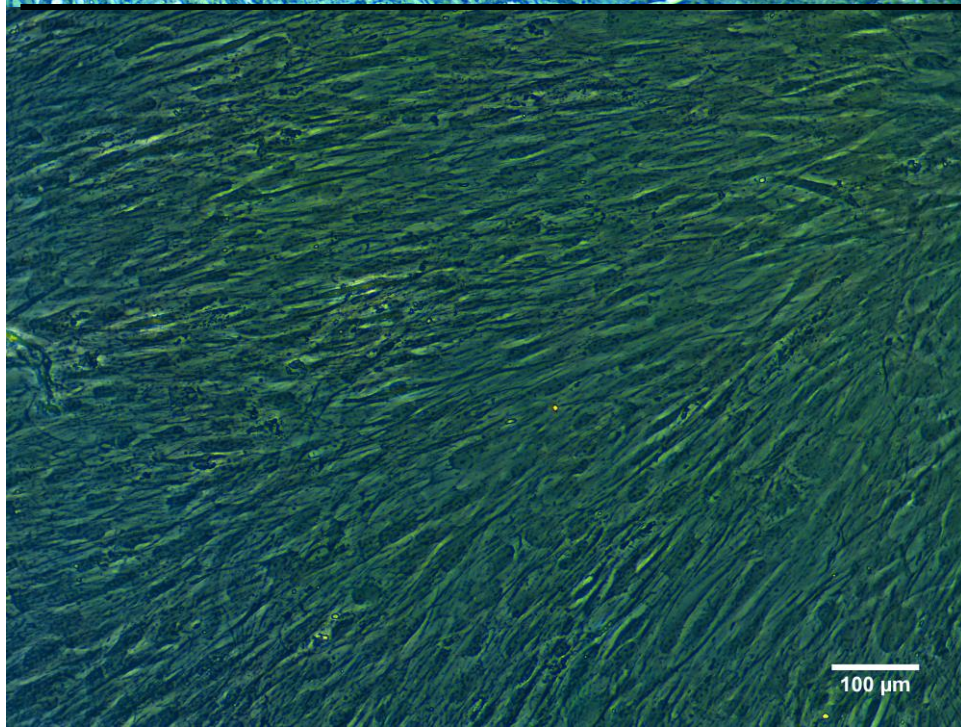**D**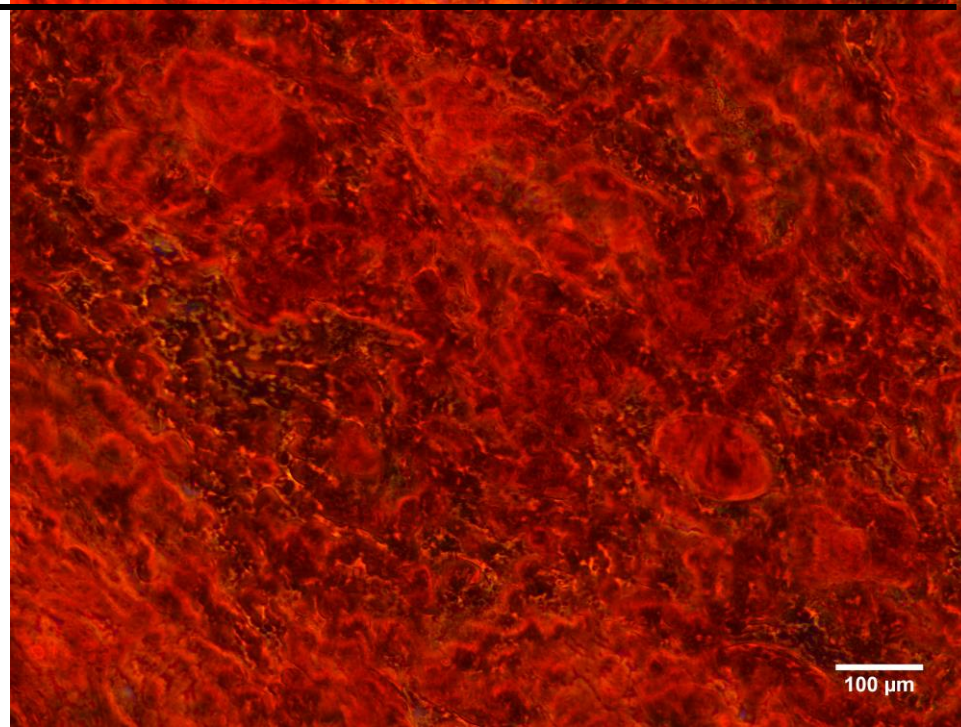

Supplement: Supplementary file 2 — Additional file 2: Figures S9-S15. fresh and cryopreserved cells stained positive for Oil Red O staining and Alizarin Red staining after incubation with adipogenic and osteogenic differentiation media. Figure S9. Oil Red O staining of fresh and cryopreserved M4 cells following 19 days of incubation in adipogenic differentiation media: Light microscopy representative images: (A) M4 fresh control well, (B) M4 fresh differentiation well, (C) M4 cryopreserved control well, (D) M4 cryopreserved differentiation well. Scale bars represent 100μm. Figure S10. Oil Red O staining of fresh and cryopreserved M6 cells following 19 days of incubation in adipogenic differentiation media: Light microscopy representative images: (A) M6 fresh control well, (B) M6 fresh differentiation well, (C) M6 cryopreserved control well, (D) M6 cryopreserved differentiation well. Scale bars represent 100μm. Figure S11. Oil Red O staining of fresh and cryopreserved M7 cells following 19 days of incubation in adipoegnic differentiation media: Light microscopy representative images: (A) M7 fresh control well, (B) M7 fresh differentiation well, (C) M7 cryopreserved control well, (D) M7 cryopreserved differentiation well. Scale bars represent 100μm. Figure S12. Alizarin Red staining of fresh and cryopreserved M4 cells following 16 days of incubation in osteogenic differentiation media: Light microscopy representative images: (A) M4 fresh control well, (B) M4 fresh differentiation well, (C) M4 cryopreserved control well, (D) M4 cryopreserved differentiation well. Scale bars represent 100μm. Figure S13. Alizarin Red staining of fresh and cryopreserved M6 cells following 16 days of incubation with osteogenic differentiation media: Light microscopy representative images: (A) M6 fresh control well, (B) M6 fresh differentiation well, (C) M6 cryopreserved control well, (D) M6 cryopreserved differentiation well. Scale bars represent 100μm. Figure S14. Alizarin Red staining of fresh and cryopreserved M7 cells f [file 13287_2020_2054_MOESM2_ESM.zip › Supplementary 13.pdf]

**A**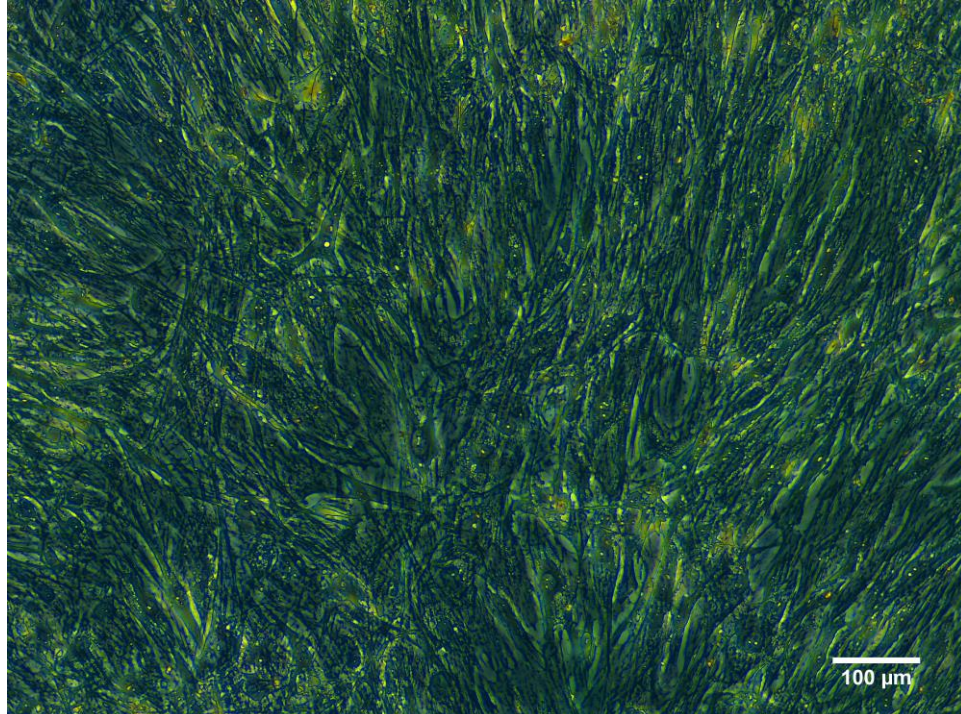**B**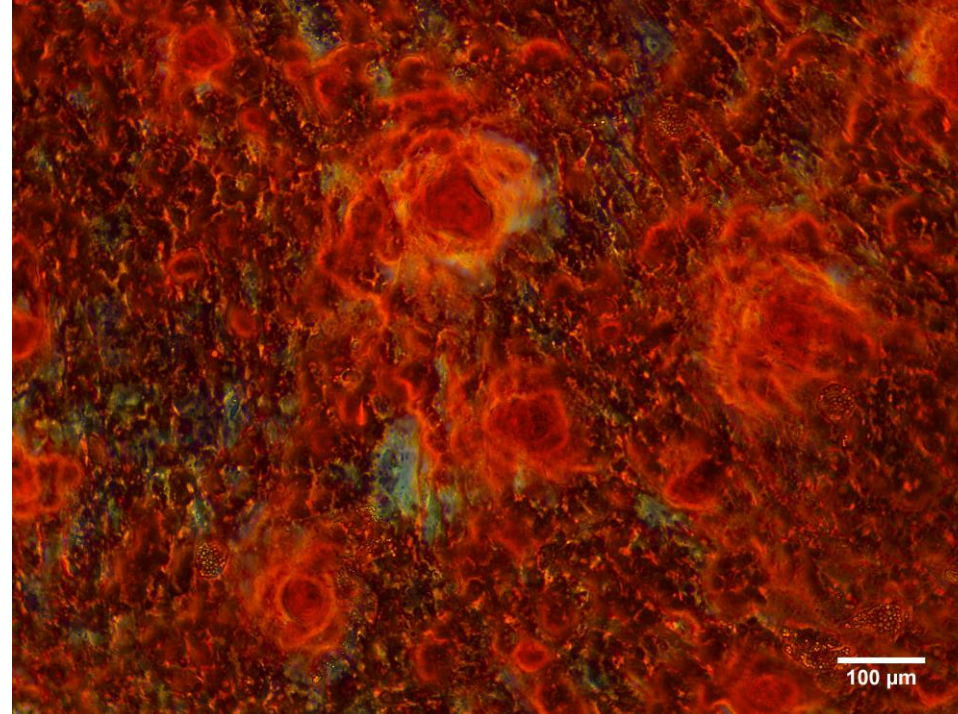**C**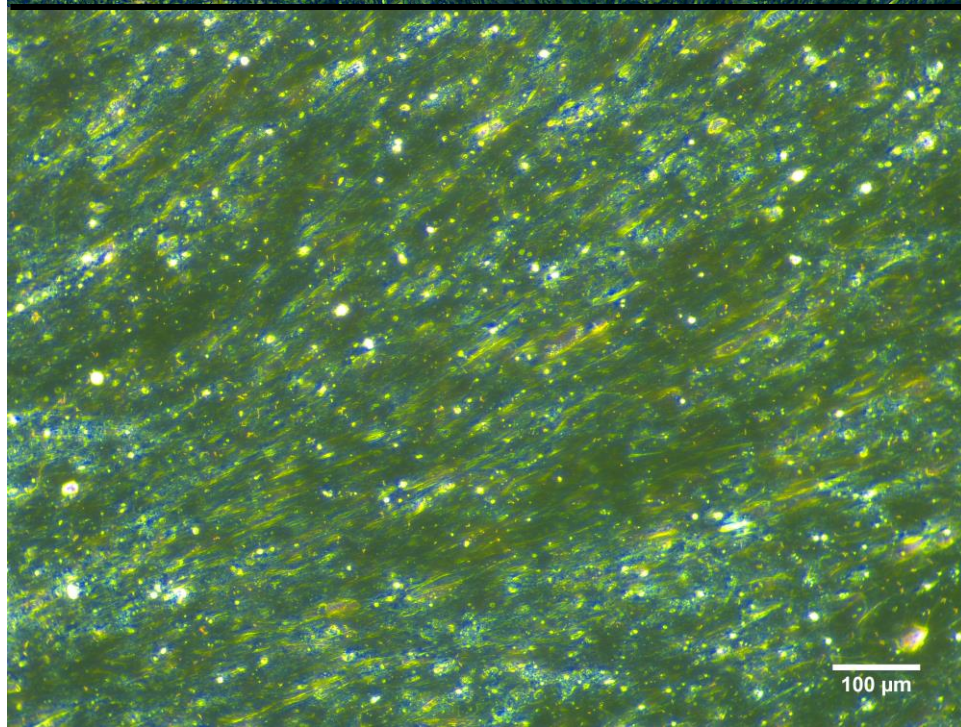**D**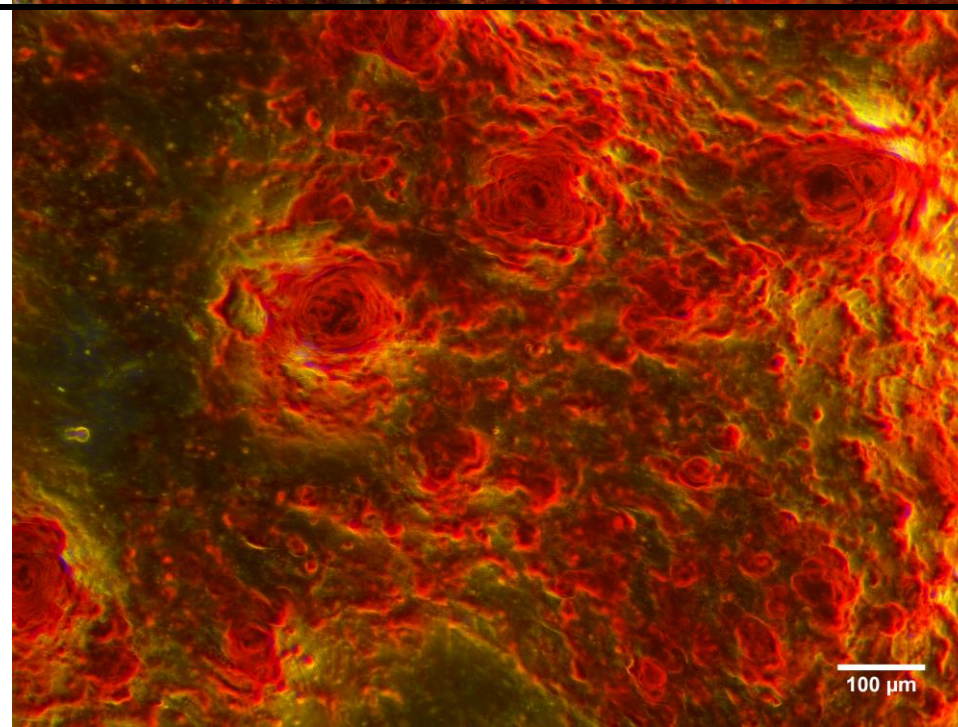

Supplement: Supplementary file 2 — Additional file 2: Figures S9-S15. fresh and cryopreserved cells stained positive for Oil Red O staining and Alizarin Red staining after incubation with adipogenic and osteogenic differentiation media. Figure S9. Oil Red O staining of fresh and cryopreserved M4 cells following 19 days of incubation in adipogenic differentiation media: Light microscopy representative images: (A) M4 fresh control well, (B) M4 fresh differentiation well, (C) M4 cryopreserved control well, (D) M4 cryopreserved differentiation well. Scale bars represent 100μm. Figure S10. Oil Red O staining of fresh and cryopreserved M6 cells following 19 days of incubation in adipogenic differentiation media: Light microscopy representative images: (A) M6 fresh control well, (B) M6 fresh differentiation well, (C) M6 cryopreserved control well, (D) M6 cryopreserved differentiation well. Scale bars represent 100μm. Figure S11. Oil Red O staining of fresh and cryopreserved M7 cells following 19 days of incubation in adipoegnic differentiation media: Light microscopy representative images: (A) M7 fresh control well, (B) M7 fresh differentiation well, (C) M7 cryopreserved control well, (D) M7 cryopreserved differentiation well. Scale bars represent 100μm. Figure S12. Alizarin Red staining of fresh and cryopreserved M4 cells following 16 days of incubation in osteogenic differentiation media: Light microscopy representative images: (A) M4 fresh control well, (B) M4 fresh differentiation well, (C) M4 cryopreserved control well, (D) M4 cryopreserved differentiation well. Scale bars represent 100μm. Figure S13. Alizarin Red staining of fresh and cryopreserved M6 cells following 16 days of incubation with osteogenic differentiation media: Light microscopy representative images: (A) M6 fresh control well, (B) M6 fresh differentiation well, (C) M6 cryopreserved control well, (D) M6 cryopreserved differentiation well. Scale bars represent 100μm. Figure S14. Alizarin Red staining of fresh and cryopreserved M7 cells f [file 13287_2020_2054_MOESM2_ESM.zip › Supplementary 14.pdf]

**A**

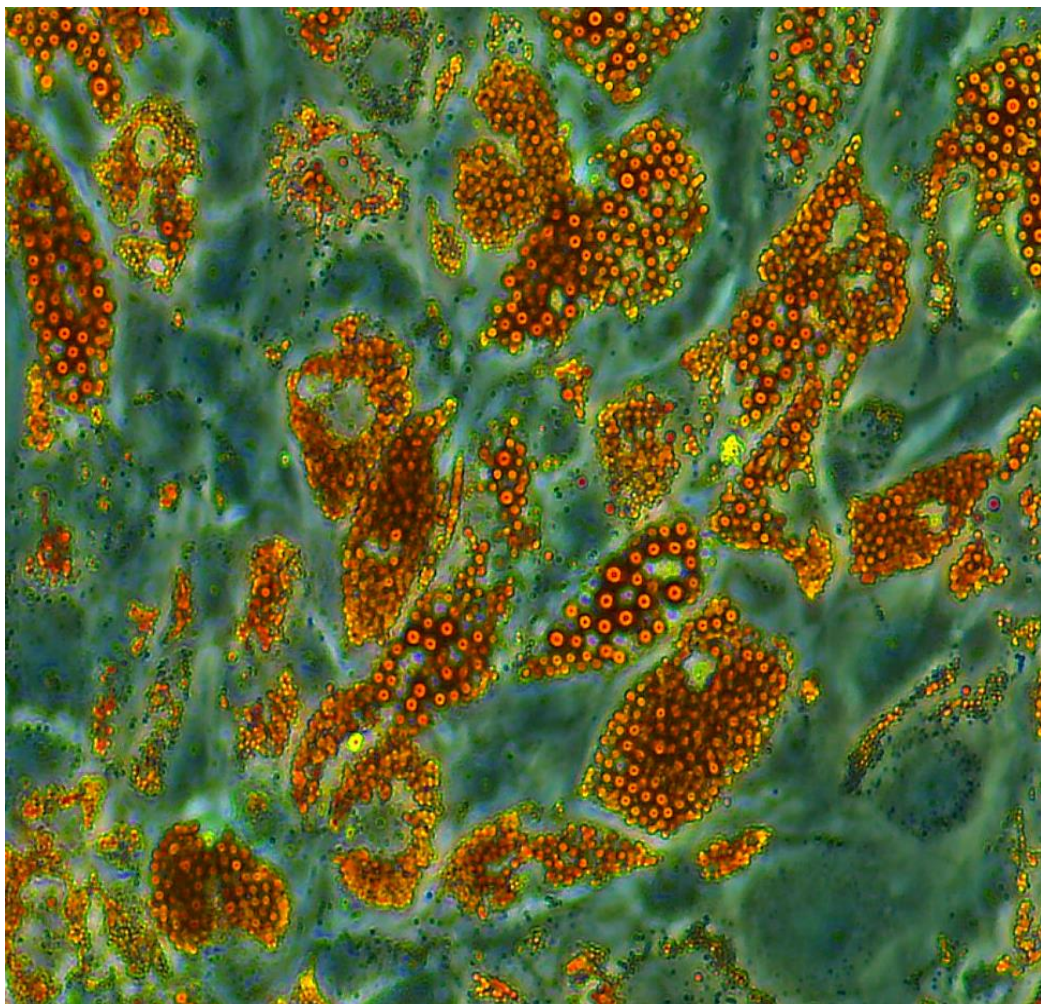

**B**

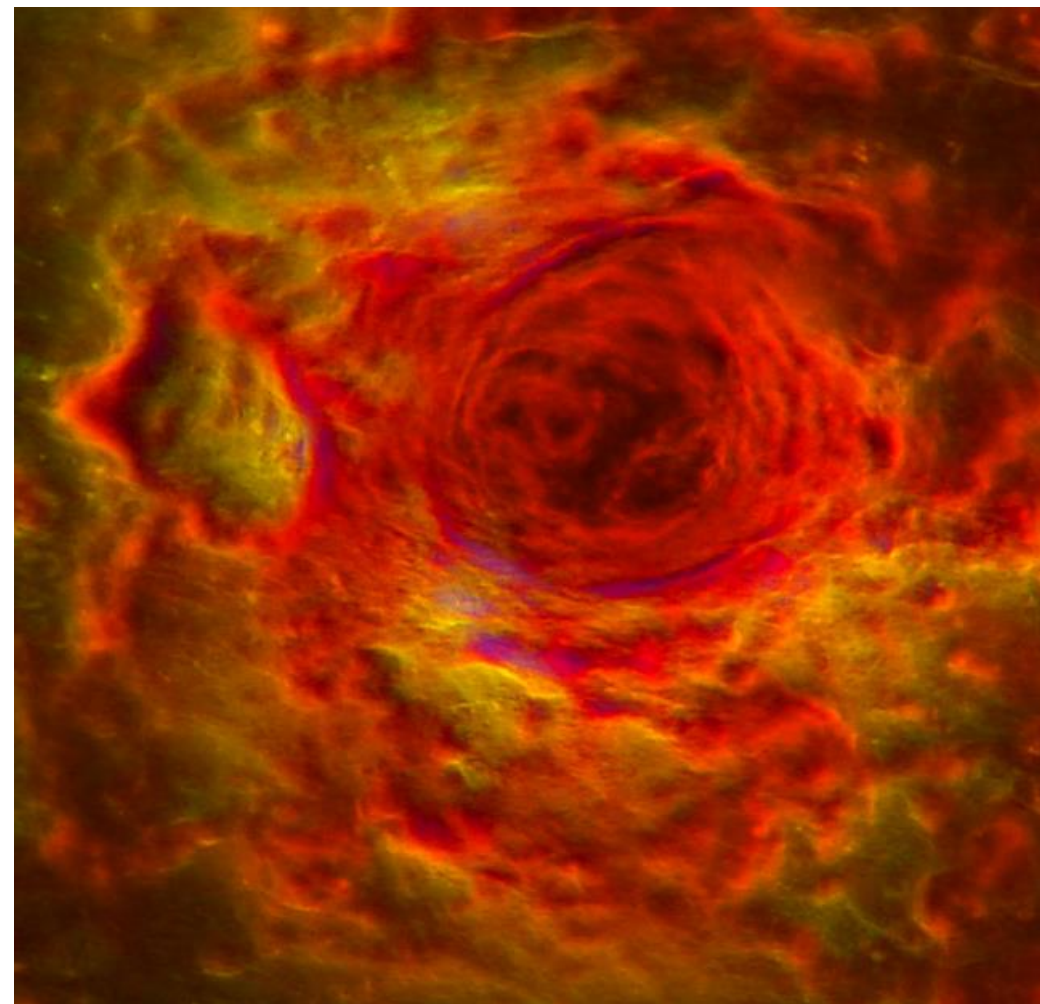

Supplement: Supplementary file 2 — Additional file 2: Figures S9-S15. fresh and cryopreserved cells stained positive for Oil Red O staining and Alizarin Red staining after incubation with adipogenic and osteogenic differentiation media. Figure S9. Oil Red O staining of fresh and cryopreserved M4 cells following 19 days of incubation in adipogenic differentiation media: Light microscopy representative images: (A) M4 fresh control well, (B) M4 fresh differentiation well, (C) M4 cryopreserved control well, (D) M4 cryopreserved differentiation well. Scale bars represent 100μm. Figure S10. Oil Red O staining of fresh and cryopreserved M6 cells following 19 days of incubation in adipogenic differentiation media: Light microscopy representative images: (A) M6 fresh control well, (B) M6 fresh differentiation well, (C) M6 cryopreserved control well, (D) M6 cryopreserved differentiation well. Scale bars represent 100μm. Figure S11. Oil Red O staining of fresh and cryopreserved M7 cells following 19 days of incubation in adipoegnic differentiation media: Light microscopy representative images: (A) M7 fresh control well, (B) M7 fresh differentiation well, (C) M7 cryopreserved control well, (D) M7 cryopreserved differentiation well. Scale bars represent 100μm. Figure S12. Alizarin Red staining of fresh and cryopreserved M4 cells following 16 days of incubation in osteogenic differentiation media: Light microscopy representative images: (A) M4 fresh control well, (B) M4 fresh differentiation well, (C) M4 cryopreserved control well, (D) M4 cryopreserved differentiation well. Scale bars represent 100μm. Figure S13. Alizarin Red staining of fresh and cryopreserved M6 cells following 16 days of incubation with osteogenic differentiation media: Light microscopy representative images: (A) M6 fresh control well, (B) M6 fresh differentiation well, (C) M6 cryopreserved control well, (D) M6 cryopreserved differentiation well. Scale bars represent 100μm. Figure S14. Alizarin Red staining of fresh and cryopreserved M7 cells f [file 13287_2020_2054_MOESM2_ESM.zip › Supplementary 15.pdf]

**A**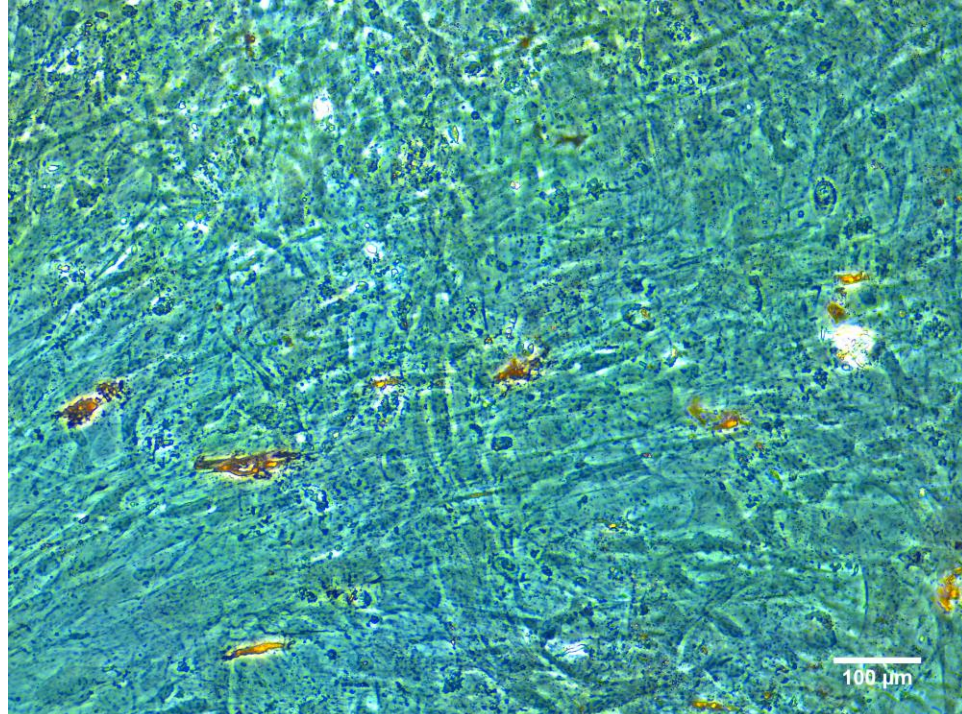**B**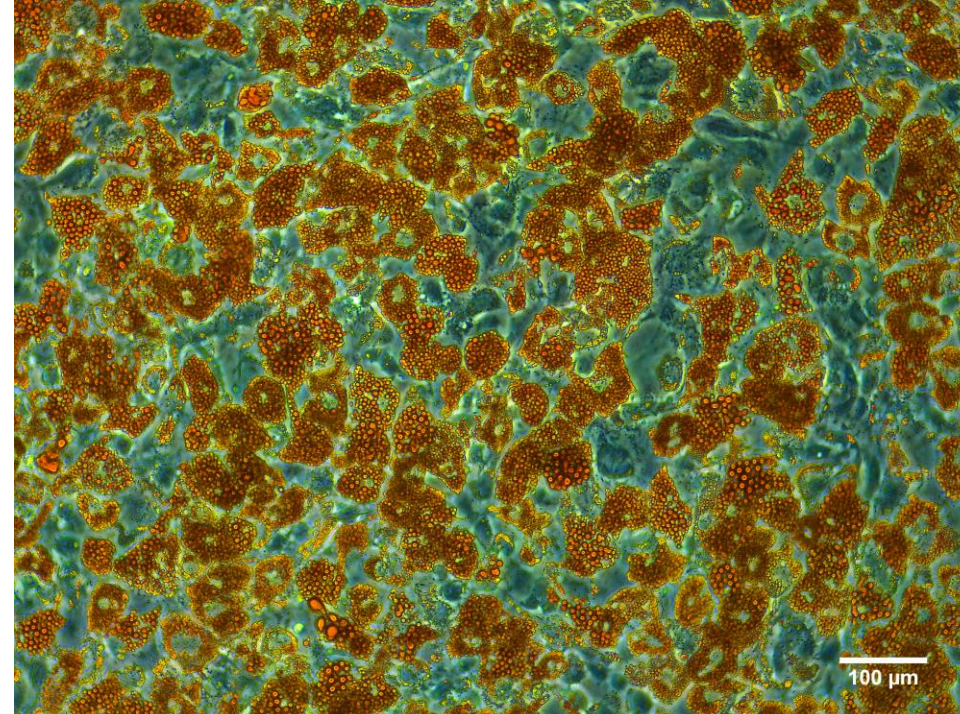**C**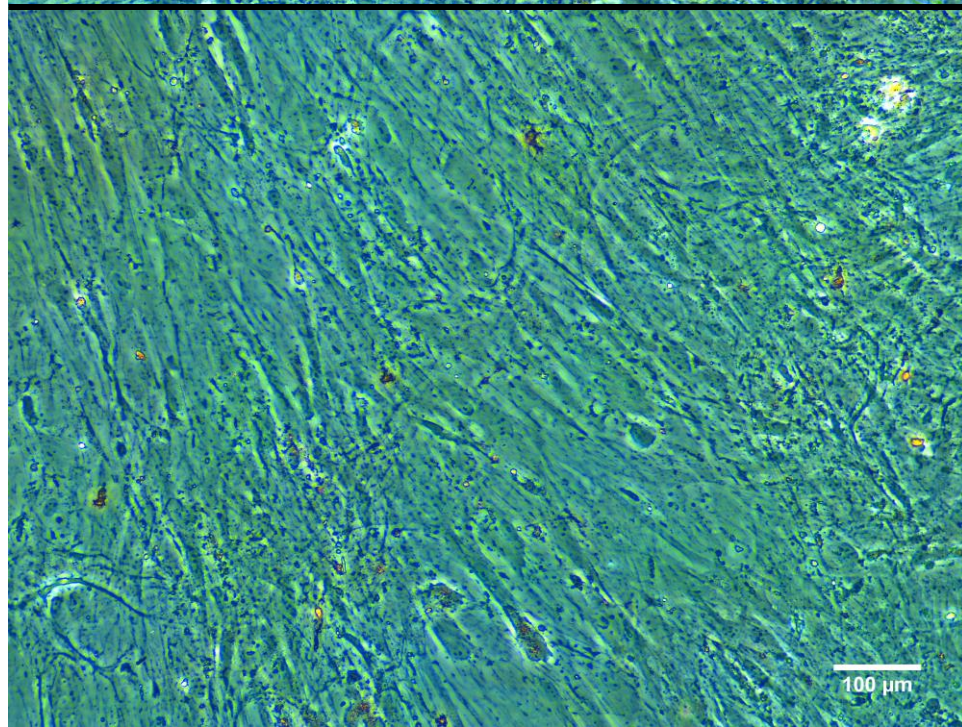**D**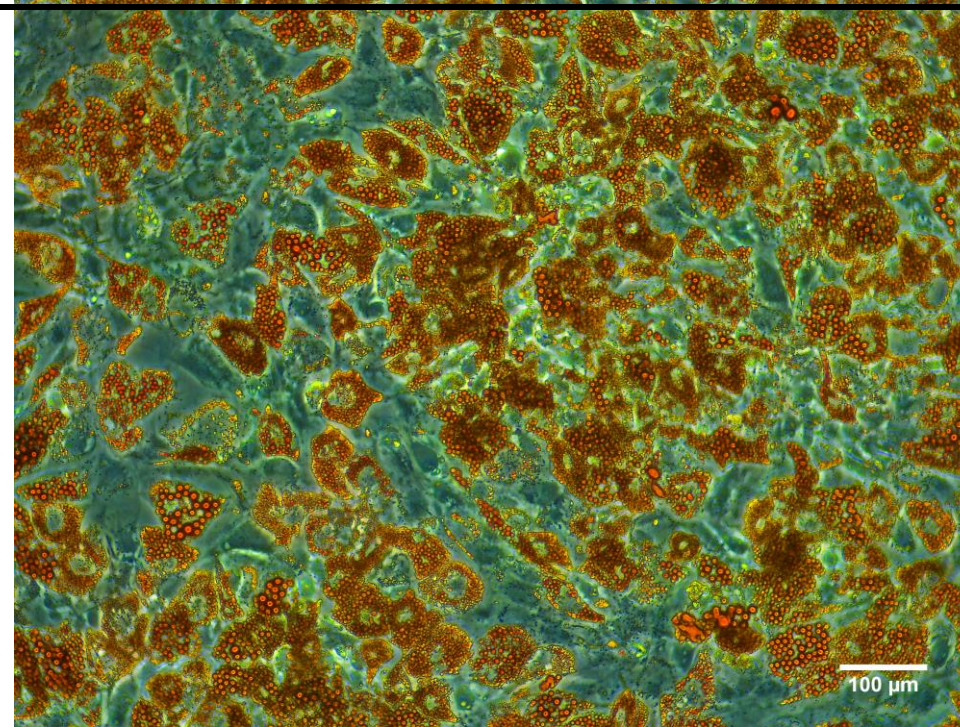

Supplement: Supplementary file 2 — Additional file 2: Figures S9-S15. fresh and cryopreserved cells stained positive for Oil Red O staining and Alizarin Red staining after incubation with adipogenic and osteogenic differentiation media. Figure S9. Oil Red O staining of fresh and cryopreserved M4 cells following 19 days of incubation in adipogenic differentiation media: Light microscopy representative images: (A) M4 fresh control well, (B) M4 fresh differentiation well, (C) M4 cryopreserved control well, (D) M4 cryopreserved differentiation well. Scale bars represent 100μm. Figure S10. Oil Red O staining of fresh and cryopreserved M6 cells following 19 days of incubation in adipogenic differentiation media: Light microscopy representative images: (A) M6 fresh control well, (B) M6 fresh differentiation well, (C) M6 cryopreserved control well, (D) M6 cryopreserved differentiation well. Scale bars represent 100μm. Figure S11. Oil Red O staining of fresh and cryopreserved M7 cells following 19 days of incubation in adipoegnic differentiation media: Light microscopy representative images: (A) M7 fresh control well, (B) M7 fresh differentiation well, (C) M7 cryopreserved control well, (D) M7 cryopreserved differentiation well. Scale bars represent 100μm. Figure S12. Alizarin Red staining of fresh and cryopreserved M4 cells following 16 days of incubation in osteogenic differentiation media: Light microscopy representative images: (A) M4 fresh control well, (B) M4 fresh differentiation well, (C) M4 cryopreserved control well, (D) M4 cryopreserved differentiation well. Scale bars represent 100μm. Figure S13. Alizarin Red staining of fresh and cryopreserved M6 cells following 16 days of incubation with osteogenic differentiation media: Light microscopy representative images: (A) M6 fresh control well, (B) M6 fresh differentiation well, (C) M6 cryopreserved control well, (D) M6 cryopreserved differentiation well. Scale bars represent 100μm. Figure S14. Alizarin Red staining of fresh and cryopreserved M7 cells f [file 13287_2020_2054_MOESM2_ESM.zip › Supplementary 9.pdf]

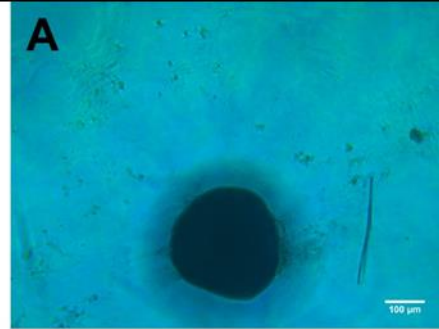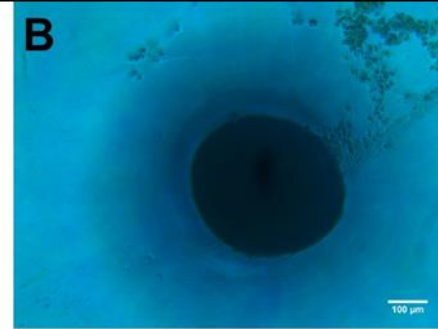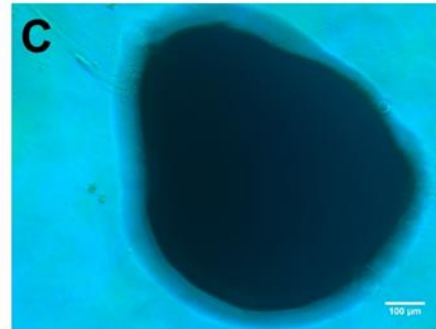

Supplement: Supplementary file 3 — Additional file 3. For chondrogenic differentiation, cells were centrifuged, and supernatant completely removed to obtain a cell pellet. 3x5μL droplets of concentrated cells were added to 12-well plate. The generated micro-masses were incubated for about an hour in a humidified incubator to allow attachment. After incubation, 1 mL/well of differentiation media was added. The differentiation media consisted of 100 nM dexamethasone, 10% ITS-Premix (Sigma, UK), 1 μg/mL ascorbic acid, 1% sodium pyruvate (Sigma, UK) and 10 ng/mL Human TGFβ1 (R&D systems, UK) in DMEM high glucose. At day 21 in culture, cells were fixed by incubating with paraformaldheyde then stained with filtered (1%w/v) Alcian Blue (Sigma, UK) in Hydrochloric acid. After one-hour incubation with the stain, wells were washed twice with distilled water and then imaged (Figure S16). Figure S16. Alcian Blue staining of fresh cells following 21 days of incubation with chondrogenic differentiation media: Representative images: (A) M4 cell line, (B) M6 cell line and (C) M7 cell line. Scale bars represent 100 μm. [file 13287_2020_2054_MOESM3_ESM.pdf]
